# *Listeria* exploits IFITM3 to suppress antibacterial activity in phagocytes

**DOI:** 10.1038/s41467-021-24982-0

**Published:** 2021-08-17

**Authors:** Joel M. J. Tan, Monica E. Garner, James M. Regeimbal, Catherine J. Greene, Jorge D. Rojas Márquez, Dustin A. Ammendolia, Adam R. R. McCluggage, Taoyingnan Li, Katherine J. Wu, Marija Cemma, Philip P. Ostrowski, Brian Raught, Michael S. Diamond, Sergio Grinstein, Robin M. Yates, Darren E. Higgins, John H. Brumell

**Affiliations:** 1 Cell Biology Program, Hospital for Sick Children, Toronto, ON Canada; 2 Institute of Medical Science, University of Toronto, Toronto, ON Canada; 3 Department of Microbiology, Blavatnik Institute, Harvard Medical School, Boston, MA USA; 4 Department of Biochemistry and Molecular Biology, University of Calgary, Calgary, AB Canada; 5 Department of Molecular Genetics, University of Toronto, Toronto, ON Canada; 6 Department of Biochemistry, University of Toronto, Toronto, ON Canada; 7 Princess Margaret Cancer Centre, University Health Network, Toronto, ON Canada; 8 Department of Medical Biophysics, University of Toronto, Toronto, ON Canada; 9 Departments of Medicine, Molecular Microbiology, Pathology & Immunology, Washington University School of Medicine, St. Louis, MO USA; 10 SickKids IBD Centre, Hospital for Sick Children, Toronto, ON Canada

**Keywords:** Interferons, Immune evasion, Bacterial infection, Innate immunity

## Abstract

The type I interferon (IFN) signaling pathway has important functions in resistance to viral infection, with the downstream induction of interferon stimulated genes (ISG) protecting the host from virus entry, replication and spread. *Listeria monocytogenes* (*Lm*), a facultative intracellular foodborne pathogen, can exploit the type I IFN response as part of their pathogenic strategy, but the molecular mechanisms involved remain unclear. Here we show that type I IFN suppresses the antibacterial activity of phagocytes to promote systemic *Lm* infection. Mechanistically, type I IFN suppresses phagosome maturation and proteolysis of *Lm* virulence factors ActA and LLO, thereby promoting phagosome escape and cell-to-cell spread; the antiviral protein, IFN-induced transmembrane protein 3 (IFITM3), is required for this type I IFN-mediated alteration. *Ifitm3*^−/−^ mice are resistant to systemic infection by *Lm*, displaying decreased bacterial spread in tissues, and increased immune cell recruitment and pro-inflammatory cytokine signaling. Together, our findings show how an antiviral mechanism in phagocytes can be exploited by bacterial pathogens, and implicate IFITM3 as a potential antimicrobial therapeutic target.

## Abstract (web summary)

Interferon (IFN) is an important component of antiviral immunity, but can also be exploited by bacteria for immune evasion. Here the authors show that *Listeria monocytogenes (Lm)* induces type I IFN to suppress the degradation of *Lm* virulence proteins, ActA and LLO, and promote *Lm* infection in an IFITM3-dependent manner, thereby hinting at a potential target for antimicrobial therapy.

## Introduction

*Listeria monocytogenes* (*Lm*) is a facultative intracellular pathogen that can cause systemic infection in mammalian hosts. After ingestion by immune cells such as macrophages, *Lm* secretes virulence factors that can lyse the bacteria-containing phagosome and escape into the cytosol^1,2^. Once in the cytosol, *Lm* is able to interact with host actin-related protein (Arp) 2/3 complex using its virulence factor actin assembly-inducing protein (ActA) to polymerize actin and form actin comet tails to propel itself throughout the cell^3^. Motility is critical for *Lm* pathogenesis as it allows the bacteria to avoid host cell autophagic machinery and the ubiquitin-proteasome system^4–6^. To spread from cell-to-cell, motile *Lm* initiate formation of cell-surface filopodia (called protrusions) that can be engulfed by neighboring cells and mediate bacterial transfer without exposure to the extracellular space^7^.

Type 1 interferons (IFNs) are a subgroup of interferon proteins that help regulate innate and adaptive immune responses during viral and bacterial infections^8–10^. *Lm* infection is able to induce a robust type I IFN response during infection. Rupture of the bacteria-containing phagosome leads to the cytosolic delivery of cyclic dinucleotides, DNA and other factors that induce type I IFN production^11–13^. *Lm* is known to benefit from the type I IFN response, as evidenced by the finding that mice deficient in type I IFN receptors and regulatory factors are protected from *Lm* infection^14–16^. However, the molecular mechanisms behind bacterial exploitation of type I IFN signaling remain unclear.

Type I IFN is known to alter many aspects of the immune response to bacteria^17^. For example, *Lm*-induced type 1 IFN production negatively regulates chemokine signaling and modulates neutrophil and monocyte recruitment^18,19^. We previously showed that type I IFN also promotes *Lm* cell-to-cell spread^20^. The early stages of actin-based motility, a process involving Arp2/3-mediated actin polymerization on the bacterial surface, were enhanced by type I IFN by mechanisms that remain unclear. As the type I IFN transcriptional response involves hundreds of genes^21^, it is also not clear which ISGs promote *Lm* infection. This led us to examine how type I IFN affects the early stages of bacterial infection in macrophages, an important cellular target of these bacteria during systemic disease.

In this study, we examine the impact of type I IFN on phagosomal maturation and bacterial killing. We identify IFITM3 as a downstream target of type I IFN activation that suppresses proteolytic activity in phagocytes, thereby promoting *Lm* cell-to-cell spread in macrophages. Cells and mice lacking IFITM3 display an increase in ActA degradation which leads to a decrease in *Lm* infection. Our study has important implications for understanding the role of type I IFN and IFITM3 in innate immunity during bacterial infections.

## Results

### Type I IFN impairs macrophage killing of phagosome-confined bacteria

**Fig. 1:**
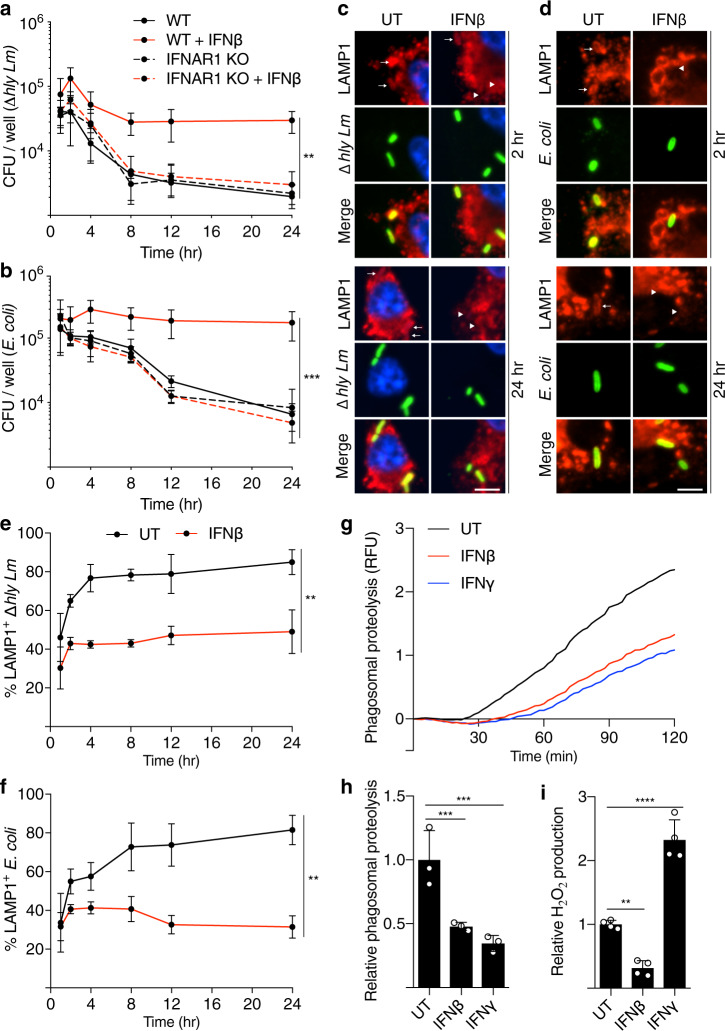
Type I IFN suppresses antibacterial activity in phagosomes. Time course of bacteria killing over 24 h of (**a**) Δ*hly Lm* or (**b**) *E. coli* infected WT and IFNAR1 KO BMDMs with or without IFNβ pretreatment. **P = 0.0012, ***P = 0.001. Representative images (**c**, **d**) and quantification (**e**, **f**) of LAMP1-positive bacteria in BMDMs treated with or without IFNβ, infected with Δ*hly Lm* (**c**, **e**) or *E. coli* (**d**, **f**) over 24 h. **P < 0.0087. Arrow denotes LAMP1-positive bacteria, arrowhead denotes LAMP1-negative bacteria. Bulk proteolysis over time (**g**) and slope of the curve’s linear portion (**h**) of DQ-BSA-labeled particles in untreated (UT) phagosomes, or treated with IFNβ or IFNγ. ***P < 0.0009. RFU relative fluorescence unit. **i** Measurement of ROS production in phagosomes of zymosan-fed BMDMs with and without IFNβ or IFNγ. **P = 0.0042, ****P < 0.0001. Data shown are means ± standard deviation (s.d.) for at least *n* = 3 independent experiments. *P* value was calculated using (**a**, **b**) two-way analysis of variance (ANOVA) and (**e**, **f**, **h**, **i**) one-way ANOVA. Scale bars, 3 µm.

To determine if type I IFN affects the antimicrobial activity of macrophages, we examined the fate of two different bacteria confined to phagosomes. We used the gram-positive *Lm* lacking the pore-forming toxin listeriolysin O (LLO, encoded by *hly*). Since listeriolysin O is required for phagosome escape in macrophages, this mutant strain is confined to phagosomes and does not undergo intracellular growth^22^. As a model of gram-negative bacteria, we also examined *Escherichia coli* DH10B, a non-pathogenic lab strain. Bone marrow-derived macrophages (BMDMs) from wild-type (WT) C57BL/6 mice were treated with interferon beta (IFNβ) for 20 hours (h) prior to exposure to bacteria. The uptake of bacteria was unaffected by IFNβ treatment (Supplementary Fig. 1a, b). The number of viable intracellular bacteria was quantified over a 24 h time course, and we observed a reduced bacterial load over time for both bacterial strains in untreated (UT) BMDMs, consistent with antibacterial activity in macrophage phagosomes (Fig. 1a, b). However, the intracellular bacterial load did not decrease in cells treated with IFNβ during this time course, though this effect was lost in IFNβ-treated BMDMs from mice lacking the type I IFN receptor (*Ifnar1*^−/−^, hereafter IFNAR1 KO). These findings suggest the type I IFN response suppresses antibacterial activity in phagosomes.

Next, we examined the impact of type I IFN on phagosome maturation. IFNβ-treated cells showed reduced localization of the lysosome transmembrane protein LAMP1 to phagosomes compared to control cells (Fig. 1c–f). This effect was readily seen at late times (after 4 h) post phagocytosis, indicating that sustained LAMP1 delivery was affected by type I IFN. However, this effect was not apparent at the earliest time points examined (1 h), indicating that early phagosome maturation events were not affected. Consistent with this finding, rapid delivery of internalized bacteria and dextran-containing endosomes to acidified late endocytic compartments, visualized by pre-loading cells with cresyl violet, was not affected by IFNβ treatment (Supplementary Fig. 2a–d). Using a real-time fluorometric FRET-based assay to measure phagosome maturation, we also found that IFNβ treatment had no effect on phagosome fusion with endocytic compartments over a 2 h time course (Supplementary Fig. 2e). These results suggest that type I IFN affects delivery of some lysosomal components to bacteria-containing phagosomes but does not affect early phagosome interactions with existing endocytic compartments.

By using fluorescence ratiometric imaging of CFSE-conjugated particles to measure the calibrated pH value of phagosomes, we determined that the rate of acidification in IFNβ-treated BMDMs was unaffected (Supplementary Fig. 1c). Similarly, we found that the steady-state pH of lysosomes measured using FITC-dextran was not affected by IFNβ treatment (Supplementary Fig. 1d). Therefore, these data indicate that type I IFN does not affect the acidification of phagosomes or lysosomes.

### Type I IFN suppresses phagosome proteolysis

Phagosomal proteases contribute to killing and digestion of internalized microbes^23^. It is known that type II IFN (IFNγ) treatment can suppress phagosomal proteolysis to promote antigen presentation^24–26^. This phenotype is mediated by the NOX2 NADPH oxidase, a multisubunit complex that generates reactive oxygen species (ROS) within the lumen of the phagosome. ROS-mediated oxidation of critical cysteine residues in phagosome-localized proteases, such as cathepsin B and S, contributes to suppression of phagosomal proteolysis^27^, along with other potential mechanisms^28^. Since IFNγ is known to promote bacterial killing^29,30^, ROS-mediated suppression of phagosomal proteolysis may be compensated by the direct antimicrobial activity of ROS^31^.

We examined phagosomal proteolytic activity in type I IFN-treated BMDMs. We utilized a real-time fluorometric assay using a dequenchable probe conjugated directly to silica beads^32^. IFNβ-treated cells showed a deficiency in phagosomal proteolysis, comparable to the effect seen with IFNγ treatment (Fig. 1g, h). This is consistent with a decrease in bacteria associated with DQ-BSA, a marker of proteolytically active compartments, in IFNβ-treated BMDMs (Supplementary Fig. 2f, g). However, cells treated with IFNβ displayed decreased ROS production following phagocytosis relative to IFNγ treatment and to untreated control cells (Fig. 1i). These findings indicate that type I IFN can suppress phagosomal proteolysis by a mechanism independent of ROS generation.

**Fig. 2:**
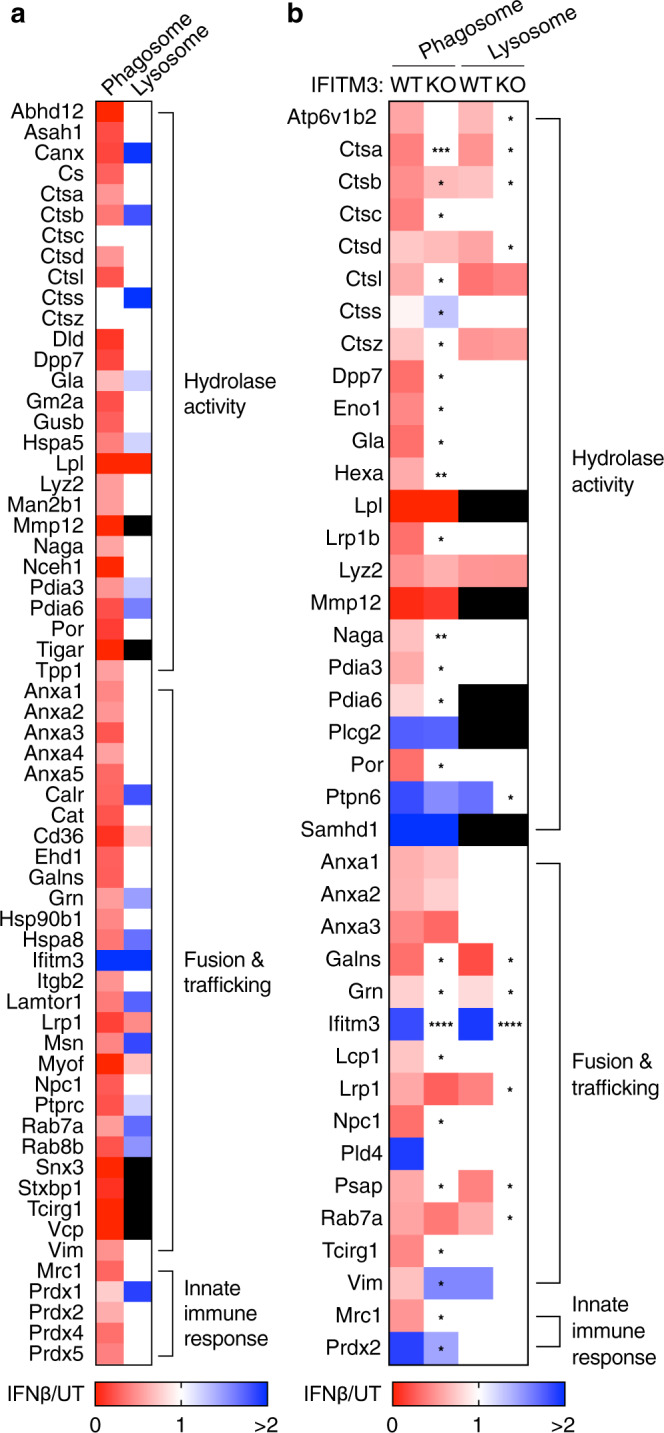
Type I IFN inhibits the delivery of hydrolase activity-related proteins to phagosomes. Proteomics data obtained by mass spectrometry of phagosomes and lysosomes isolated from (**a**) WT BMDMs and (**b**) IFITM3 KO BMDMs. Legend denotes relative protein abundance between IFNβ treatment and UT controls. Black box indicates undetected proteins. Data shown are means for at least *n* = 3 independent experiments. *P* value was calculated using two-way ANOVA. *P < 0.05, **P < 0.01, ***P < 0.001, ****P < 0.0001.

To further determine the effects of type I IFN on phagosome proteolysis, we examined the phagosome proteome using mass spectrometry. Phagosomes were isolated from IFNβ-treated BMDMs using magnetic latex beads. In separate experiments, we isolated lysosomes using iron dextran. We observed that proteolytic enzymes, as well as fusion and trafficking factors, were decreased in phagosomes from IFNβ-treated BMDMs (Fig. 2a). In contrast, lysosomal protein contents were largely unaffected by IFNβ treatment. This data suggests that type I IFN inhibits the delivery of proteolytic enzymes to the phagosome during the maturation process.

### Type I IFN inhibits phagosomal proteolysis of *Lm* virulence factors

Phagosomal proteases can degrade bacterial virulence factors, limiting their ability to alter host cell machinery^33^. Therefore, we hypothesized that type I IFN-mediated suppression of phagosomal proteolysis would lead to enhanced activity of bacterial virulence factors within this compartment. During WT *Lm* infection, a number of virulence factors, including LLO and two phospholipases C, act cooperatively to modify phagosome maturation and promote bacterial escape into the cytosol^1^. ActA also acts cooperatively with these virulence factors in the phagosome. ActA is a bacterial cell surface-associated virulence factor that was originally identified for its role in actin-based motility of *Lm* in the cytosol of host cells - a requirement for direct cell-to-cell spread by these bacteria^34^. Further studies revealed a role for ActA in promoting phagosome escape^35^.

**Fig. 3:**
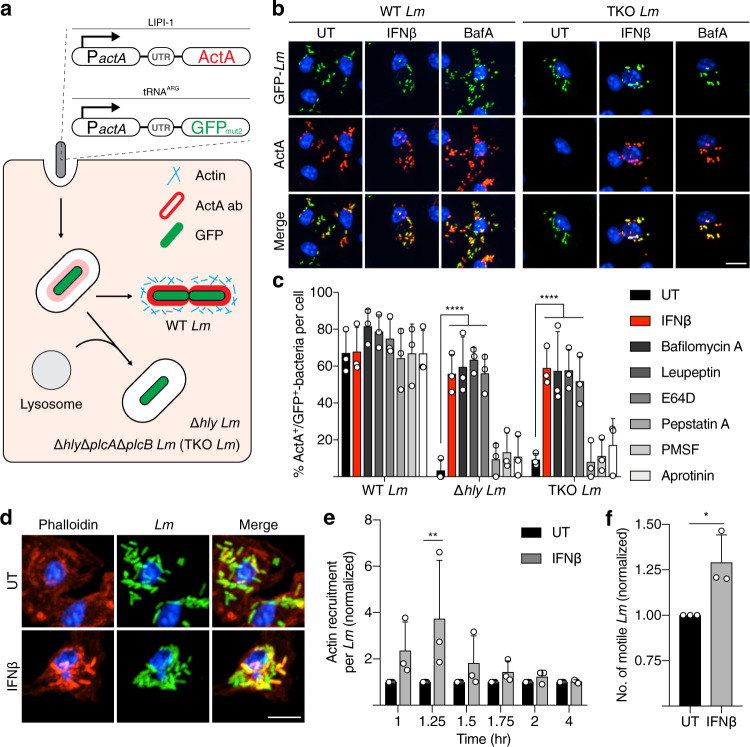
Type I IFN suppresses ActA degradation in phagosomes and promotes *Lm* actin recruitment. **a** Schematic of GFP reporter *Lm* strains developed to measure *actA* transcription activation. Representative images (**b**) and quantification (**c**) of *actA*:GFP reporter expression and ActA degradation of BMDMs infected for 4 h with indicated *Lm* strains measured by GFP fluorescence and antibody staining, with or without treatment of IFNβ, bafilomycin A or protease inhibitors. Representative images at 1.25 hr p.i. (**d**) and quantification (**e**) of WT *Lm* actin recruitment, measured by phalloidin staining, after phagosome escape over 4 h time course. **f** Measurement of WT *Lm* actin-based motility with phalloidin-positive comet tails 4 h p.i. Data shown are means ± s.d. for *n* = 3 independent experiments. *P* value was calculated using (**c**, **e**) two-way ANOVA and (**f**) two-tailed unpaired *t*-test. *P = 0.0285, **P = 0.001, ****P < 0.0001. Scale bars, 11 µm.

To visualize ActA expression and cell surface localization during infection we generated fluorescent reporter strains (Fig. 3a, Supplementary Fig. 3a). Transcription of *actA* was detected by green fluorescence protein (GFP) expression under the *actA* promoter, in a cassette stably integrated in the tRNA^ARG^ locus of the bacterial chromosome. This cassette contains all of the known enhancer elements for ActA expression^36^ and we used the enhanced mutant of GFP for optimal detection of *actA* transcription (Supplementary Fig. 3a). The endogenous copy of *actA* within *Listeria* Pathogenicity Island 1 (LIPI-1) was left intact. Bacteria surface-localized ActA was detected by immunofluorescence using affinity-purified antibodies. The *actA*:GFP reporter strain was generated in the WT *Lm* background as well as the Δ*hly* mutant and a mutant lacking virulence factors required for phagosomal escape (Δ*hly*, Δ*plcA* and Δ*plcB*), hereafter referred to as the triple knockout (TKO) *Lm* strain (Fig. 3a). ActA expression was previously thought to be initiated only after bacterial escape from the phagosome^37,38^. However, we observed that *Lm* expression of ActA can occur within phagosomes of J774 macrophages, prior to escape into the cytosol (Supplementary Fig. 3b).

In control BMDMs and RAW264.7 cells infected with the WT *actA*:GFP reporter strain, the majority of bacteria were localized to the cytosol at 4 h post-infection (p.i.), and displayed robust transcription of *actA*, visualized by the expression of *actA*:GFP and localization of ActA protein on the bacterial cell surface, as expected (Fig. 3b, Supplementary Fig. 3d, e). In contrast, Δ*hly* and TKO *Lm* trapped in LAMP1-positive phagolysosomes lacked surface ActA, despite high levels of GFP fluorescence, indicative of active *actA*:GFP expression (Supplementary Fig. 3d, f). We considered the possibility that ActA is degraded by proteases in phagosomes under these conditions. In support of this hypothesis, treatment of cells with broad-spectrum protease inhibitors promoted surface expression of ActA in phagosome-trapped bacteria (Fig. 3c). Furthermore, inhibition of phagosomal acidification and proteolysis with the vacuolar-type proton ATPase inhibitor bafilomycin A1^39^, had a similar effect (Fig. 3b, c, Supplementary Fig. 3c). Our findings indicate that ActA is actively degraded by phagosomal proteases prior to bacterial escape into the cytosol under normal infection conditions.

**Fig. 4:**
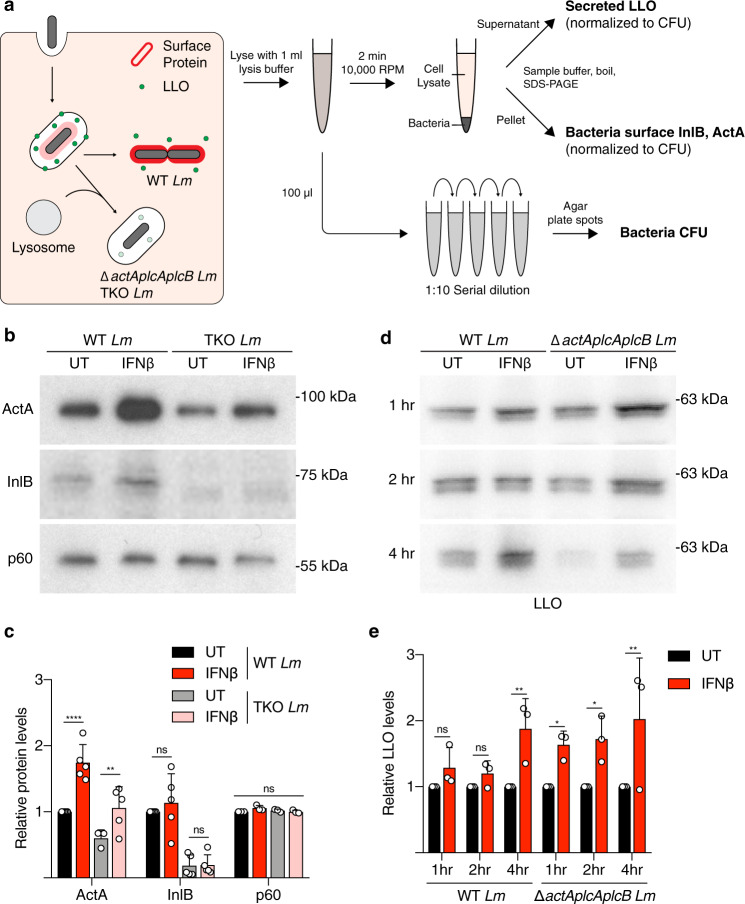
Type I IFN suppresses *Lm* virulence factor degradation in phagosomes. **a** Schematic of experimental protocol detailing methods to obtain secreted and bacterial lysate fractions from BMDMs infected with indicated *Lm* strains. Representative images (**b**) and quantification (**c**) of ActA and InlB on the bacteria surface measured by western blot in BMDMs infected with indicated *Lm* strains 4 h p.i. Cell wall-associated protein p60 used as loading control. **P = 0.002, ****P < 0.0001. Representative images (**d**) and quantification (**e**) of LLO measured by western blot in BMDMs infected with indicated *Lm* strains over 4 h. *P < 0.0295, **P < 0.0039. Data shown are means ± standard deviation (s.d.) for at least *n* = 3 independent experiments. *P* value was calculated using two-way ANOVA. ns not significant, *P* > 0.1301. Source data are provided as a Source Data file.

Next, we examined the impact of type I IFN treatment on ActA expression during *Lm* infection. BMDMs were treated with IFNβ prior to infection with *actA*:GFP reporter strains (for immunofluorescence imaging) and WT and TKO *Lm* strains (for protein analysis). Under these conditions, ActA localized to the bacterial surface in phagosome-trapped bacteria (Δ*hly* and TKO *Lm*) (Fig. 3b, c), suggesting that type I IFN-mediated suppression of phagosomal proteolysis stabilizes ActA. To further examine virulence factor expression during infection, we utilized a cell fractionation approach that separates bacterial cell surface proteins from secreted proteins (Fig. 4a). Western blotting revealed higher levels of ActA protein in lysates from cells treated with type I IFN (Fig. 4b, c). We also examined InlB, a bacterial surface protein required for invasion of host cells^40^. Levels of InlB dropped in the TKO *Lm* strain compared to WT bacteria, consistent with degradation of InlB by phagosomal proteases. However, levels of InlB were unchanged in IFNβ-treated BMDMs infected with WT and TKO *Lm* (Fig. 4b, c). These findings indicate that type I IFN selectively modulates phagosomal proteolysis of bacterial surface-associated proteins.

We also explored the impact of type I IFN on secreted LLO. For these studies, we used a triple mutant of *Lm* lacking other phagosome escape factors (Δ*actA*, Δ*plcA,* and Δ*plcB*) to decrease the efficiency of bacterial entry to the cytosol. Secreted LLO was isolated from the detergent soluble fraction of cell lysates isolated after *Lm* infection (Fig. 4a). In IFNβ-treated BMDMs, secreted LLO was elevated in the Δ*actAplcAplcB Lm* strain at 1, 2 and 4 h p.i. (Fig. 4d, e). We also observed higher levels of secreted LLO at 4 h p.i. by WT *Lm*. We conclude that type I IFN suppresses phagosomal proteolysis of *Lm* virulence factors required for phagosome escape.

Since ActA and LLO contribute to phagosome escape^22,35^, we examined the impact of type I IFN treatment on this process. IFNβ treatment led to an increase in actin recruitment to WT *Lm* during the early stages of bacterial spread (Fig. 3d, e), concomitant with a higher number of motile *Lm* with actin comet tails at 4 h p.i. (Fig. 3f). These findings are consistent with our prior study showing that type I IFN promotes *Lm* cell-to-cell spread^20^, possibly through a positive feedback mechanism between actin polymerization and comet tail formation previously described^41^. Together, these results reveal that type I IFN-mediated suppression of phagosomal proteolysis can promote the activity of *Lm* virulence factors that regulate intracellular infection and dissemination of these bacteria.

### IFITM3 promotes *Lm* cell-to-cell spread

To enter the cytosol during infection of host cells, some viruses exploit proteases during the viral fusion process. For example, severe acute respiratory syndrome coronavirus (SARS-CoV) and SARS-CoV-2 utilizes host type II transmembrane serine proteases and cathepsin L to infect cells expressing its ACE2 receptor^42–44^. Conformational changes in the viral S glycoprotein mediated by proteolysis are thought to enable viral fusion. Similarly, endosomal proteolysis is required for infection by Ebola virus, influenza A and rotaviruses^45–47^.

Interferon-induced transmembrane protein 3 (IFITM3) is an antiviral ISG in mice and humans that localizes to cell surface and endocytic compartments in most cell types during type I IFN activation^48–50^. Consistent with this notion, we observed IFITM3 to be the only fusion and trafficking-associated protein strongly localizing to both phagosomes and lysosomes in IFNβ-treated BMDMs (Fig. 2a). IFITM3 mediates viral resistance by blocking fusion between virus and host membranes^51^, and by inhibiting viral envelope processing and capsid protein proteolysis^52,53^. IFITM3 is also localized to bacteria-containing phagosomes during *Legionella pneumophila* infection^54^. Based on these findings we evaluated whether IFITM3 contributes to type I IFN-mediated changes in phagocyte function during *Lm* infection.

**Fig. 5:**
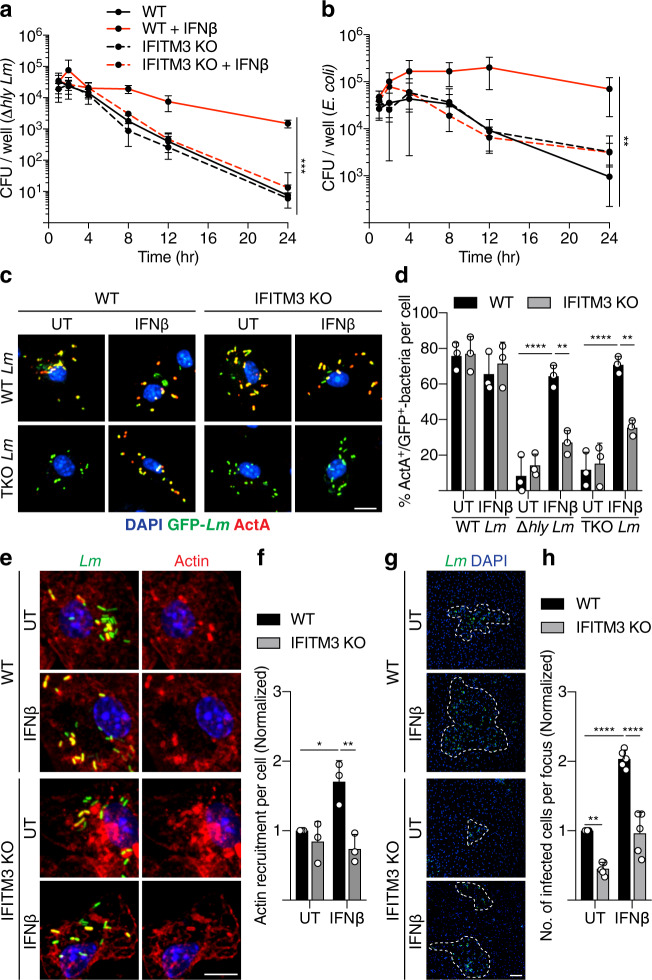
IFITM3 suppresses antibacterial activity in phagosomes and promotes *Lm* cell-to-cell spread. Time course of bacteria killing over 24 h of (**a**) Δ*hly Lm* or (**b**) *E. coli* infected WT and IFITM3 KO BMDMs with or without IFNβ. Representative images (**c**) and quantification (**d**) of ActA degradation of IFITM3 WT and KO BMDMs infected for 4 h with indicated *actA*:GFP reporter *Lm* strains measured by antibody staining of surface ActA under treatment with or without IFNβ. Scale bar, 11 µm. Representative images (**e**) and quantification (**f**) of WT *Lm* actin recruitment after phagosome escape at 1.25 h p.i. in IFITM3 WT and KO BMDMs treated with or without IFNβ. Scale bar, 11 µm. Representative images (**g**) and quantification (**h**) of an infection focus assay to measure WT *Lm* cell-to-cell spread. WT and IFITM3 KO BMDMs were treated with or without IFNβ and infected for 18 h. Dotted lines delineate edge of the infection foci. Scale bar, 70 µm. Data shown are means ± s.d. for at least *n* = 3 independent experiments. *P* value was calculated using two-way ANOVA. *P = 0.0242, **P < 0.0041, ***P = 0.001, ****P < 0.0001.

BMDMs were isolated from *Ifitm3*^−/−^ mice (hereafter IFITM3 KO) and infected with phagosome-confined bacteria. We observed a reduced bacterial load over time for both Δ*hly Lm* and *E. coli* in untreated BMDMs from WT and IFITM3 KO mice, consistent with antibacterial activity in macrophage phagosomes (Fig. 5a, b). However, the type I IFN-mediated defect in antibacterial activity was not observed in IFITM3 KO BMDMs. Thus, IFITM3 contributes to suppression of killing of phagosome-confined bacteria following type I IFN treatment.

We also examined the role of IFITM3 in suppressing phagosomal proteolysis. Type I IFN-mediated accumulation of ActA on the surface of phagosome-confined *actA*:GFP reporter *Lm* (Δ*hly* and TKO) was lost in IFITM3 KO BMDMs (Fig. 5c, d). Similarly, type I IFN-mediated accumulation of secreted LLO in WT and Δ*actAplcAplcB Lm*-infected BMDMs was lost in IFITM3 KO cells (Supplementary Fig. 4a–c). Next, we looked at early proteolysis dynamics in live cells using DQ-BSA labeled beads. We found that the decrease in proteolysis of IFNβ-treated BMDMs was not affected by the loss of IFITM3 (Supplementary Fig. 5a, b). We also performed proteomic analysis of phagosomes isolated from IFITM3 KO BMDMs after 4 h. Loss of IFITM3 was sufficient to revert some of the IFNβ-mediated alterations to proteins responsible for hydrolytic activity and fusion and trafficking events (Fig. 2b). Lysosomal protein content was mostly unaffected by IFNβ treatment (Fig. 2b), suggesting a decrease in proteolysis only in the phagosome. Finally, we quantified and found a type I IFN-mediated increase in actin recruitment in WT *Lm*-infected BMDMs, an effect that was lost in IFITM3 KO cells (Fig. 5e, f). Together, these findings indicate that IFITM3 selectively modulates phagosomal proteolysis and bacteria phagosome escape following type I IFN treatment.

Next, we examined the impact of IFITM3 on cell-to-cell spread of WT *Lm* by performing an infection focus assay^7^. We show that IFITM3 KO BMDMs displayed a decrease in *Lm* spread (Fig. 5g, h). We also found that IFNβ-treated BMDMs displayed increased *Lm* cell-to-cell spread in an IFITM3-dependent manner. The ability of *Lm* to grow in the cytosol was unaffected by IFNβ or the loss of IFITM3 (Supplementary Fig. 5c). These results suggest that expression of IFITM3 plays a significant role in promoting *Lm* cell-to-cell spread in BMDMs.

### IFITM3 promotes *Lm* infection in vivo

**Fig. 6:**
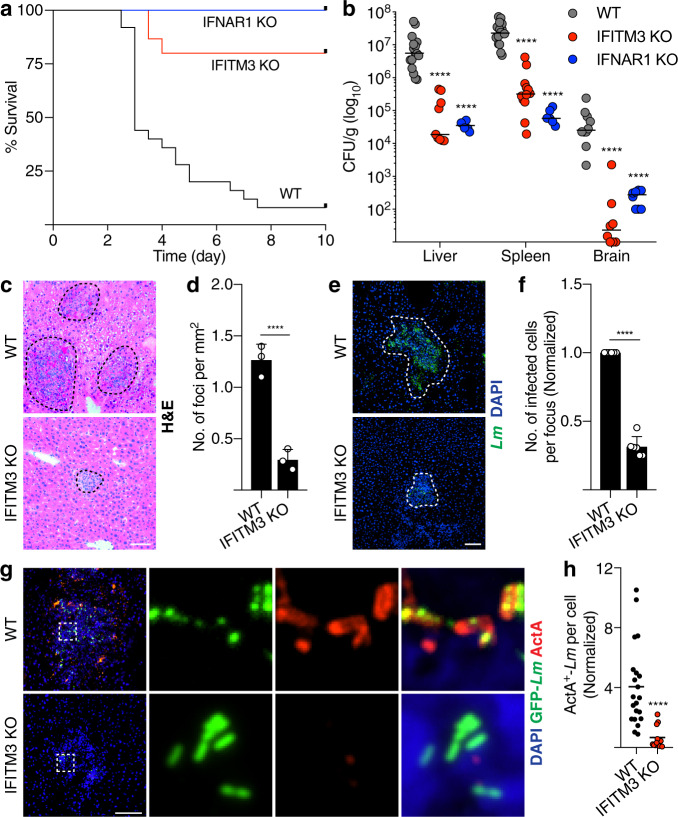
IFITM3 promotes *Lm* infection in vivo. **a** 10-day survival rate of WT (*n* = 25), IFITM3 (*n* = 15), and IFNAR1 KO (*n* = 10) mice infected with WT *Lm* plotted using Kaplan–Meier function. **b** Bacteria load of liver, spleen and brain of WT (*n* ≥ 10), IFITM3 (*n* ≥ 8), and IFNAR1 KO (*n* ≥ 6) mice infected with *Lm* over 72 h. Representative images (**c**, **e**) and quantification (**d**, **f**) of infection foci taken from the liver of mice (*n* = 3) 72 h p.i. Tissues were stained with H&E (**c**, **d**) or *Lm* and DAPI (**e**, **f**). Dotted lines delineate edge of the infection foci. Representative images (**g**) and quantification (**h**) of cell surface ActA expression by *Lm* in liver taken from mice (*n* = 3) 72 h p.i. Data shown are medians (**b**, **h**) or means ± s.d. (**d**, **f**). *P* value was calculated using two-tailed Mann–Whitney test (**b**, **f**) and two-tailed unpaired *t*-test (**d**, **f**). ****P < 0.0001. Scale bars, 70 µm.

Next, we examined the impact of IFITM3 on *Lm* infection in vivo. WT and IFITM3 KO mice were inoculated by tail vein injection with WT *Lm* to examine 10-day survival, as well as to obtain tissue homogenates from the liver, spleen, and brain to measure bacterial load after 72 h. IFITM3 KO mice were protected against *Lm* infection, with increased survival rates after 10 days compared to WT mice (Fig. 6a). IFITM3 KO mice also displayed a significant decrease in bacteria harvested from the liver, spleen and brain compared to control animals (Fig. 6b). Consistent with this, liver sections from IFITM3 KO mice revealed a decrease in total number of infection foci per square mm of liver tissue (Fig. 6c, d), as well as a decrease in the number of infected cells per infection focus (Fig. 6e, f), compared to WT mice. Similar changes were observed in IFNAR1 KO mice (Fig. 6a, b), consistent with previous findings^14^. We also found higher numbers of ActA-positive *Lm* in WT compared to IFITM3 KO livers (Fig. 6g, h), consistent with a decrease in proteolysis of this virulence factor seen in vitro. Together, this data suggests that IFITM3 is a key downstream target of type I IFN signaling that *Lm* exploits to promote its dissemination.

**Fig. 7:**
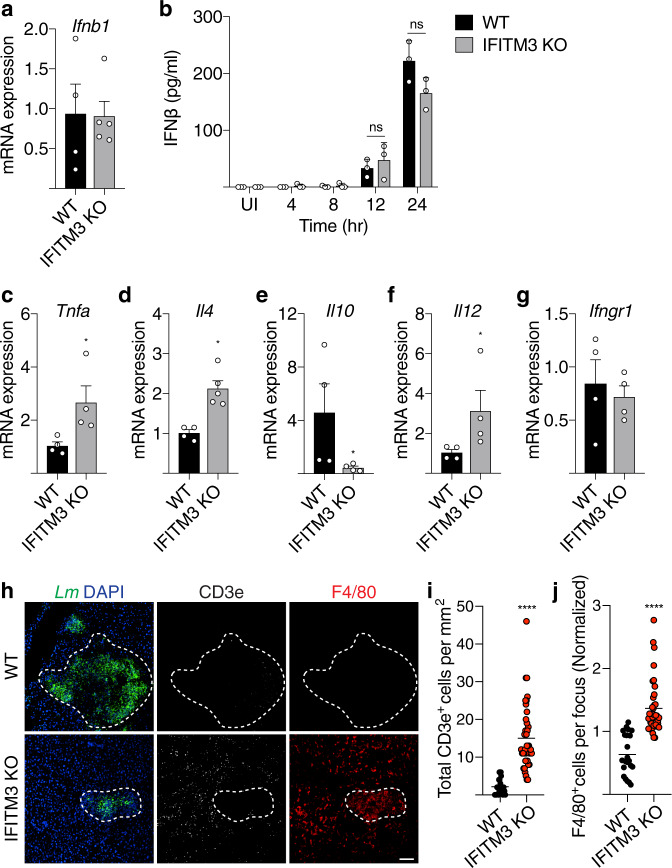
IFITM3 modulates cytokine signaling and immune cell recruitment. **a** mRNA expression of IFNβ from *Lm*-infected liver of mice (*n* ≥ 4) 72 h p.i. **b** IFNβ production measured by ELISA of WT and IFITM3 KO BMDMs infected with *Lm* over 24 h. UI uninfected. **c**–**g** mRNA expression of indicated cytokines from *Lm*-infected liver of mice (*n* ≥ 4) 72 h p.i. Representative images (**h**) and quantification of (**i**) lymphocyte recruitment (CD3e) and (**j**) macrophage recruitment (F/480) of liver taken from mice (*n* = 3) 72 h p.i. Dotted lines delineate edge of the infection foci. Data shown are means (**a**–**g**) or medians (**i**, **j**) ± s.d. for at least *n* = 3 independent experiments. *P* value was calculated using two-tailed unpaired *t*-test (**a**–**g**) and two-tailed Mann–Whitney test (**i**, **j**). *P < 0.05, ****P < 0.0001. ns not significant, *P* > 0.055. Scale bar, 70 µm.

IFITM3 deficiency has previously been shown to display higher levels of inflammatory cytokines during a viral infection^55^. Therefore, we examined the role of IFITM3 in cytokine production and immune cell recruitment during *Lm* infection. We found no change in levels of IFNβ production in *Lm*-infected WT and IFITM3 KO mice and BMDMs (Fig. 7a, b). In IFITM3 KO mice we observed an increase in expression of pro-inflammatory cytokines such as TNFα, IL-4 and IL-12, and reduced expression of the anti-inflammatory cytokine IL-10 (Fig. 7c–f). This effect was not due to a change in IFN gamma receptor expression (Fig. 7g). IFITM3 KO mice also displayed higher levels of lymphocyte and macrophage recruitment to sites of infection, as judged by CD3e and F4/80 staining of infected tissues, respectively (Fig. 7h–j). Thus, IFITM3 also promotes systemic infection of *Lm* in mice by suppressing immune cell recruitment and pro-inflammatory cytokine signaling.

## Discussion

**Fig. 8:**
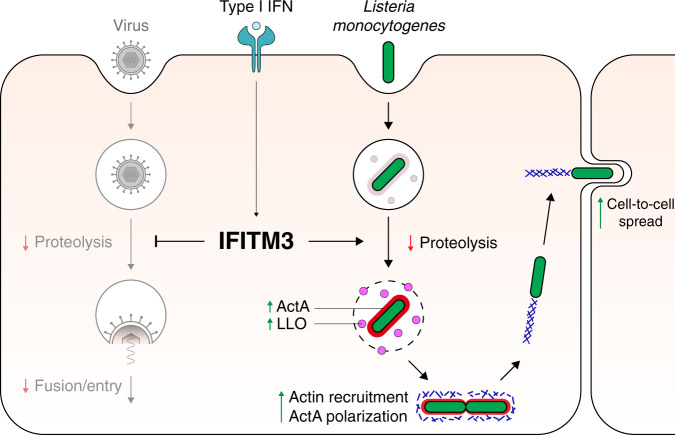
Model of type I IFN-mediated effects of IFITM3. During viral infection, IFITM3 decreases proteolysis of virus capsid proteins, and restricts fusion and entry into the cytosol. During *Lm* infection, IFITM3-mediated suppression of proteolysis leads to increased LLO and ActA in the phagosome. After entering the cytosol, increased actin recruitment subsequently leads to an increase in cell-to-cell spread. Arrows indicate decrease (red) or increase (green) by IFITM3.

In this study, we show that type I IFN can suppress the antibacterial activities of phagocytes through the actions of IFITM3. This ISG has previously been shown to selectively modulate the activity of gamma-secretase, a protease complex^56^, playing an important part in regulating protease activity within the endosomal pathway. We show that IFITM3 can selectively suppress proteolysis of some phagosomal cargoes such as ActA and LLO, leading to increased *Lm* cell-to-cell spread (Fig. 8). Our findings provide important insight into the expression and function of ActA, which was previously thought to be expressed exclusively in the cytosol^37,38^, while also revealing the impact of IFITM3 expression on this virulence factor in vivo during *Lm* infection.

IFITM3 likely benefits *Lm* infection through multiple mechanisms. The bacteria are known to secrete LLO during *Lm* protrusion formation to induce damage at the plasma membrane and promote cell-to-cell spread using a mechanism that resembles efferocytosis^7^. Membrane repair at the site of protrusion is key in preventing bacterial cell-to-cell spread by limiting exofacial PS exposure^57^. In the presence of type I IFN, macrophages and lymphocytes are sensitized to membrane damage mediated by LLO toxin by mechanisms that remain unclear^58^. It is possible that IFITM3 may be playing a separate role in promoting *Lm* cell-to-cell spread by suppressing lysosome-mediated membrane repair.

Our results also reveal a role of IFITM3 in regulating immune cell recruitment and cytokine signaling in infected tissues, though the mechanisms for this remain unclear. Type I IFN was shown to induce T-lymphocyte apoptosis through the actions of LLO^14,59,60^. Whether IFITM3 affects T-lymphocyte apoptosis to promote *Lm* infection is an important question for future studies. It is noteworthy that IFITM3 was recently shown to modulate B cell activation through promoting PI3-kinase signaling^50^. IFITM3 has also been implicated in a wide range of diseases such as cancer metastasis, tuberculosis susceptibility, and ulcerative colitis^61–63^. Our findings are consistent with an emerging view that IFITM3 is involved in many biological processes, beyond its established role in antiviral immunity.

The importance of type I IFN during bacterial infections is highlighted by its significant role in many other host-pathogen interactions^64^. Failure to clear *Streptococcus pneumoniae* and *Staphylococcus aureus* in alveolar macrophages after a viral infection is attributed to IFN-induced alteration of the innate and adaptive immune response^65,66^. Type I IFN and IFITM3 have also been found to promote *Salmonella enterica* serovar Typhimurium infection in epithelial cells by increasing lysosomal acidification and proteolysis^67^. However, this effect may be cell-type specific as mice infected with *S*. Typhimurium and other non-cytosolic pathogens are unaffected by a loss of IFITM3^68^. Consistent with this notion, we found that type I IFN treatment does not promote *Lm* spread in non-phagocytic cells such as fibroblasts and Caco-2, whereas spread is decreased in hepatocytes (Supplementary Fig. 6a–f). In future studies, it will be important to determine if other bacterial pathogens that undergo cytosolic growth and cell-to-cell spread can also exploit IFITM3 during infection in phagocytes. Since IFITM3 appears to drive much of the deleterious effects of type I IFN on the host to benefit *Lm* during infection, it represents a possible therapeutic target for the treatment of this class of bacterial pathogens.

## Methods

### Bacterial strains

*Listeria monocytogenes* (*Lm*) were grown in brain-heart infusion (BHI) broth and the following strains were used: 10403S (WT), 10403S (dasherGFP-WT), DP-L2161 (Δ*hly*), DH-L1137 (GFP-Δ*hly*), DP-L2319 (Δ*hly*Δ*plcA*Δ*plcB*, TKO), and DH-1458 (Δ*actA*Δ*plcA*Δ*plcB*). *Escherichia coli* were grown in lysogeny broth (LB) and the following strains were used: DH10B (WT) and DH10B (RFP). *E. coli* was cultured overnight, diluted to an OD_600_ of 1.0 and opsonized with IgG (Meridian) for 30 min at 37 °C prior to infection.

The following reporter strains of *Lm* expressing GFP under the *actA* promoter were used: DH-L1245 (WT), DH-L2011 (Δ*hly*) and DH-L2012 (Δ*hly*Δ*plcA*Δ*plcB*, TKO). To generate the reporter stains, the reporter construct P_*actA*_-*actA* UTR-*gfp*_mut2_ was generated by overlap extension PCR^69^. Primer 432 (CGCGGCCGGTGGTATCCCGAATAAAGCAGCC, annealing 262 bp upstream of the *actA* transcriptional start site) and primer 434 (GAAAAGTTCTTCTCCTTTACTCATTTATACTCCCTCCTCGTGATAC) were used to PCR amplify the *actA* promoter and UTR from the *Lm* chromosome. Primers 435 (GTATCACGAGGAGGGAGTATAAATGAGTAAAGGAGAAGAACTTTTC) and 221 (AAGTCGACTTATTTGTATAGTTCATCCATGCCATG) were used to PCR amplify the *gfp*_mut2_ gene from plasmid pDH-1038^70^. The two PCR products were gel purified using a QIAquick gel extraction kit (Qiagen), and the purified products were then used as templates for splicing by overlap extension PCR^69^ along with primers 432 and 221. The resulting P_*actA*_-*actA* UTR-*gfp*_mut2_ fragment was gel purified, digested with EagI and SalI, and ligated into pPL3 digested with the same restriction enzymes. The ligation was then dialyzed and electroporated into *E. coli* XL1-Blue to generate strain DH-E1244. The resulting plasmid (pDH-1244) was also electroporated into *Lm* 10403S to yield DH-L1245, *Lm* DP-L2161 to yield DH-L2011, and *Lm* DP-L2319 to yield DH-L2012. Antibiotics were used at the following concentrations: chloramphenicol 20 μg ml^−1^ for selection of pPL3-derived plasmids in *E. coli* and chloramphenicol 7.5 μg ml^−1^ for selection of single-copy pPL3-derived plasmids integrated at the tRNA^ARG^ locus in *Lm*. All primers used are listed in Supplementary Table 1.

### Animals

B6.129S2-*Ifnar1*^−/−^ (IFNAR1 KO; Jackson stock #32045-JAX) and B6(Cg)-*Tyr*^*c-2J*^-*Ifitm3*^−/−^ (IFTIM3 KO; obtained from M. Diamond, backcrossed >10 generations with C57BL/6J at Washington University at St. Louis^71^) mice were bred in-house at The Hospital for Sick Children Animal Care Facility. C57BL/6J (stock #000664) and B6(Cg)-*Tyr*^*c-2J*^ (stock #000058) mice, originally from The Jackson Laboratory, were also bred in-house and used as controls. All experiments were performed with 6–12-week-old sex- and age-matched mice that were maintained on a 12 h light–dark cycle, with food and water available ad libitum. All animals were bred separately and housed in specific pathogen-free barrier rooms kept at 22–26 °C and 45–55% humidity. Experiments were not blinded, and mice were not randomized. All experiments described in this study were carried out in accordance with the Guide for the Humane Use and Care of Laboratory Animals and ethical approval was obtained from The Hospital for Sick Children’s Animal Care Committee (AUP #52111).

### Antibodies, constructs, and reagents

Primary antibodies (1:100) used for immunofluorescence were rabbit anti-*Lm* (#B223021 from BD Biosciences), rabbit-anti-*E. coli* (#ab20640 from Abcam), rabbit-anti-ActA^72^ (gift from P. Lauer, Aduro BioTech), rabbit-anti-InlB (gift from M. Loessner, ETH Zurich), mouse-anti-p60 (#NBP2-80120 from Novus), rabbit-anti-LLO (#ab200538 from Abcam), rat-anti-F4/80 (#ab16911 from Abcam), biotin-anti-CD3e (#13-0031-85 from ThermoFisher), and rat anti-mouse LAMP1 (1D4B was deposited to the Developmental Studies Hybridoma Bank by J.T. August). Primary antibodies (1:1000) used for western blotting were rabbit-anti-ActA, rabbit-anti-InlB (gift from M. Loessner, ETH Zurich), rabbit-anti-LLO (#ab200538 from Abcam). Alexa Fluor-568/647 Phalloidin and all fluorescent secondary antibodies (Alexa Fluor conjugates) were from Invitrogen. IFNβ (#8234-MB-010; 2400 IU ml^−1^) was from R&D Systems and IFNγ (#315-05; 100 U ml^−1^) was from PeproTech. 4,6-Diamidino-2-phenylindole (DAPI) (#D1306 from Invitrogen) was used at 1:2,500 dilution to stain the nuclei where indicated. Green DQ-BSA (#D12050; 10 μg ml^−1^) and Oregon Green Dextran (#D7173; 40 μg ml^−1^) was from ThermoFisher. Cresyl violet (#C5042; 1 µM) and FITC-dextran (#FD10S) was from Sigma. Bafilomycin A (#11038; 100 nM) was from Cayman Chemical. The following protease inhibitors were from BioShop: Leupeptin (#LEU001.25; 200 μM), PMSF (#PMSF123; 1 mM), Aprotinin (#APR600.100; 1.5 μM), Pepstatin A (#PEP605.10; 10 μM). E64D (#E8640; 100 μM) was from Sigma.

### Cell culture and macrophage generation

RAW264.7 and J774 macrophages, AML12 hepatocytes, mouse embryonic fibroblasts (MEFs), and Caco-2 cells were cultured in DMEM (Hyclone) supplemented with 10% heat-inactivated FBS (Wisent) without antibiotics at 37 °C and 5% CO_2_. All cells used were authenticated and tested negative for mycoplasma by The Hospital for Sick Children Biobank and the Mycoplasma Plus PCR kit (Aligent Technologies #302008). Primary macrophages were obtained from mice housed in The Hospital for Sick Children Animal Care Facility. Mice were euthanized by CO_2_ inhalation. Mouse BMDMs were obtained from the dissected femurs and tibias of 6–8-week-old male C57BL/6 mice. Cells were washed with growth medium and plated on 70 cm^2^ Petri dishes (ThermoFisher #FB0875712). Medium was replaced every 2-3 days and after 7 days cells were used for experiments. Cells were maintained in high-glucose RPMI-1640 medium (Wisent #350-025-CL) containing 10% heat-inactivated FBS (Wisent), 1% sodium pyruvate (Wisent), 1% non-essential amino acids (Wisent), 0.5% 2-mercaptoethanol (Gibco), 1% penicillin and streptomycin (Invitrogen), and 10% L929-conditioned media while differentiating, and in RPMI and 10% FBS subsequently. L929-conditioned media was generated by growing a confluent layer of L929 cells in 175 cm^2^ flasks in DMEM supplemented with 10% heat-inactivated FBS. When cells reached confluency, growth medium was replaced with DMEM alone. After 7 days, supernatant was collected, centrifuged, filtered and stored at −20 °C.

### Immunofluorescence

Immunostaining was conducted as previously described^57^. Briefly, cells were fixed with 2.5% paraformaldehyde (PFA) for 10 min at 37 °C, permeabilized and blocked in PBS+ (Wisent) containing 0.2% saponin (Calbiochem) and 10% normal goat serum (SS-PBS) for 30 min. Subsequently, cells were incubated for 1 h with primary antibodies in SS-PBS. Cells were washed three times with PBS+ and incubated with secondary Alexa Fluor conjugated antibodies in SS-PBS for 1 h. Cells were washed three times with PBS+, mounted in fluorescence mounting medium (Dako). Confocal microscopy images were imported into Adobe Photoshop and assembled in Adobe Illustrator for labeling.

### Western blotting

Samples were normalized to bacterial CFU and subjected to SDS-PAGE using 12% polyacrylamide gels (Invitrogen #XP00125BOX) and transferred onto PVDF membranes (BioRad #1620177). Membranes were blocked using 5% non-fat dried milk diluted in TBS-Tween for 1 h. Primary and HRP-secondary antibodies were diluted at 1:1000 and 1:5000 respectively. Membranes were detected using West Pico PLUS chemiluminescent substrate (ThermoFisher # 34580) on a Bio-Rad ChemiDoc system. Densitometry was performed using ImageJ software. All uncropped and unprocessed scans of blots can be found in the Source Data File.

### Bacteria killing assay

WT, IFNAR1 KO, and IFITM3 KO BMDMs were plated at 3 × 10^5^ cells per well in 24-well tissue culture plates and treated with 2400 IU ml^−1^ IFNβ 24 h before infection. Cells were infected with Δ*hly Lm* or *E. coli* at an MOI of 1 or 1:200 respectively in RPMI. At 60 min p.i., all cells were washed three times with PBS+ and cultured in RPMI medium containing 10% FBS, 50 μg ml^−1^ gentamicin (Wisent #400-130-IG) and IFNβ. At 1, 2, 4, 8, 12, and 24 h p.i., cells were lysed with PBS+ containing 1% Triton X-100. Serial dilutions of the lysates were plated on LB- (*E. coli*) and BHI- (Δ*hly Lm*) agar plates and incubated at 37 °C for 16 h for subsequent quantification of intracellular colony forming units (CFUs).

### Phagocytosis assay

BMDMs were plated at 3 × 10^5^ cells per well in 24-well tissue culture plates and treated with 2400 IU ml^−1^ IFNβ 24 h before infection. Cells were infected with Δ*hly Lm* or *E. coli* at an MOI of 1 or 1:200 respectively in RPMI. At 60 min p.i., all cells were washed three times with PBS+ and cultured in RPMI medium containing 10% FBS, 50 μg ml^−1^ gentamicin and IFNβ. At 5, 10, 15, 20, 25 and 30 min p.i., cells were fixed with 2.5% PFA. Differential antibody staining was carried out to stain extracellular bacteria before permeabilizing the cells to stain for total bacteria. Cells were imaged on a Quorum spinning disk confocal scan head (Leica DMI 6000 B inverted fluorescence microscope, Hamamatsu ORCA Flash 4 sCMOS and color camera) equipped with a ×63 objective. Phagocytosis was measured by calculating internalized bacteria (total bacteria minus extracellular bacteria) over the time course using Volocity 6 software.

### LAMP1 recruitment

BMDMs were plated at 3 × 10^5^ cells per well in 24-well tissue culture plates and treated with 2400 IU ml^−1^ IFNβ 24 h before infection. Cells were infected with Δ*hly Lm* or *E. coli* at an MOI of 1 or 1:200 respectively in RPMI. At 60 min p.i., all cells were washed three times with PBS+ and cultured in RPMI medium containing 10% FBS, 50 μg ml^−1^ gentamicin and IFNβ. At 1, 2, 4, 8, 12, 24 h p.i., cells were fixed with 2.5% PFA. Cells were stained with antibodies against *Lm, E. coli* and LAMP1. Cells were then imaged on a Quorum spinning disk confocal scan head equipped with a ×63 objective. LAMP1 recruitment was measured by counting LAMP1-positive bacteria over the time course.

### Cresyl violet assay

BMDMs were plated at 1 × 10^5^ cells per well in µ-Slide 8 Well IBIDI chambers (#80826) and treated with 2400 IU ml^−1^ IFNβ 24 h before infection. Cells were infected with GFP-Δ*hly Lm* at an MOI of 5 in RPMI. At 60 min p.i., all cells were washed three times with PBS+ and cultured in RPMI medium containing 10% FBS, IFNβ and 50 μg ml^−1^ gentamicin. Cells were pulsed with cresyl violet (1 μM) for 5 min to label acidified compartments^73^, and washed prior to imaging and replaced with RPMI medium containing 10% FBS and IFNβ. 4 h p.i., cells were imaged live on a Quorum spinning disk confocal scan head (Leica DMI6000B inverted fluorescence microscope, Hamamatsu ImagEM ×2 camera) equipped with a ×63 objective. Cresyl violet-positive bacteria were quantified using Volocity 6 software.

### Compartment fusion assay

BMDMs were plated at 1 × 10^5^ cells per well in µ-Slide 8 Well IBIDI chambers and treated with 2400 IU ml^−1^ IFNβ 24 h before treatment. 15 min prior to cresyl violet treatment, cells were pulsed with Oregon Green 10 kDa dextran (40 µg ml^−1^) and washed. Cells were pulsed with cresyl violet (1 μM) for 5 min and washed prior to imaging and replaced with RPMI medium containing 10% FBS and IFNβ. Cells were imaged live using a spinning disk confocal microscope equipped with a ×63 objective. Pearson correlation of dextran-positive and cresyl violet-positive compartment colocalization were quantified using Volocity 6 software.

### Phagosome assay preparation

BMDMs were plated at 1.2 × 10^5^ cells per well in 96 well μClear plates (Greiner Bio-One) and treated with 100 U ml^−1^ IFNγ or 2400 IU ml^−1^ IFNβ 20 h prior to assay. Fluorescently labeled, IgG-coupled 3-μm silica particles were prepared and used for phagosomal lumenal characterization as previously detailed^74,75^. Measurements were performed in microplate format with the use of a FLUOstar Optima fluorescent plate reader (BMG Labtech) or Envision 2104 Multilabel Reader (PerkinElmar) at 37 °C. Immediately prior to the addition of experimental particles, growth medium was removed from cells and replaced with assay medium (DPBS containing 1 mM CaCl_2_, 2.7 mM KCl, 0.5 mM MgCl_2_, 5 mM glucose, 0.1% calf skin gelatin, and 10 mM HEPES). Particles were added to BMDMs at an MOI of 2-3 particles per cell.

### Phagolysosomal fusion

BMDMs were prepared as above. To determine the rate of phagosome fusion with lysosomes, a FRET-based assay was used wherein the acceptor fluorophore (Alexa Fluor 594 hydrazide) was pulsed into lysosomes overnight prior to phagocytosis of the particle restricted donor fluorophore (Alexa Fluor 488 SE). The assay was carried out in a microplate format in an Envision plate reader. The FRET signal (emission of the acceptor fluorophore following excitation of the donor fluorophore) was detected over time following phagocytosis and expressed relative to the donor fluorophore fluorescence.

### Phagosome acidification

BMDMs were prepared as above. Phagosomal pH was calculated by recording the ratio of the fluorescent emission at 520 nm of carboxyfluorescein succinimidyl ester (CFSE) conjugated to experimental particles excited at 490 nm and 450 nm followed by polynomial regression to a standard curve as previously detailed^75,76^.

### Phagosome proteolysis

BMDMs were prepared as above. For assessment of the hydrolytic capacity of the phagosome, experimental particles were prepared bearing the proteolytic substrate, green DQ-BSA and the calibration fluorophore (Alexa Fluor 594 SE) as previously described^75,77^. The hydrolytic capacity of the cells was determined as the rate of substrate liberation of the DQ-Green-BSA from the experimental particles over time, relative to the calibration fluorophore. For comparison of hydrolytic capacities across experiments, the slopes (as described by the equation *y* = mx + c, where *y* = RFU, *m* = slope, and *x* = time) of the linear portion of the relative substrate fluorescence plotted against time were calculated relative to the internal control indicated.

### Phagosome ROS production

BMDMs were prepared as above. To determine the amount of H_2_O_2_ generated by macrophages stimulated with 50 μg ml^−1^ of serum-opsonized zymosan (Sigma Aldrich), supernatant analysis was carried out by measuring the reaction of 20 μM Amplex Red (Invitrogen #A12222) in the presence of horse radish peroxidase using a FLUOstar Optima fluorescence plate reader (BMG Labtech) as previously described^77,78^. Hydrogen peroxide production is presented as relative to unstimulated macrophages.

### Phagosome and lysosome isolation

WT and IFITM3 KO BMDMs were plated at 1 × 10^7^ cells per dish in 70 cm^2^ Petri dishes and treated with 2400 IU ml^−1^ IFNβ for 24 h prior to and throughout the experiment. 7 × 10^7^ cells (7 dishes) and 4 × 10^7^ cells (4 dishes) were used per condition for phagosome and lysosome isolation respectively. For phagosomes, magnetic latex beads (2.79 µm, Bangs Laboratories #UMC3001-UMC3N) were opsonized with IgG for 60 min at 37 °C. Beads were diluted in RPMI + 10% FBS and fed to BMDMs at a ratio of 10 beads per cell. Beads were allowed to phagocytose for 30 min before washing cells with PBS+ and replacing with RPMI + 10% FBS for 4 h. For lysosomes, 5 ml media containing 2.5 ml iron dextran (FeDex, obtained as a gift from R. Yates) in H_2_O mixed with 2.5 ml 2X RPMI + 10% FBS, was added to 5 ml regular RPMI + 10% FBS, to make a total volume of 10 ml used per dish. Cells were pulsed for 8 h with the FeDex solution before washing with PBS+ and chased by replacing with RPMI + 10% FBS for 1 h.

Subsequent steps were performed on ice or in 4 °C. To isolate phagosomes and lysosomes, cells were washed twice with cold PBS+, replaced with 7 ml cold PBS+, scraped, and collected in a 50 ml tube. Cells were spun down at 450 × *g* for 5 min and pellet was resuspended in 10 ml homogenization buffer (250 mM sucrose, 3 mM imidazole, pH 7.4) and spun down again. Final cell pellets were resuspended in 2.5 ml homogenization buffer and homogenized using a 15 ml Dounce homogenizer with a small clearance pestle for 20–30 passes until 90% of cells were broken without nuclear breakage. Homogenate were spun down in 15 ml tube at 135 × *g* for 5 min and supernatant was collected in a 5 ml propylene round bottom tube. To purify phagosomes and lysosomes, tubes with supernatant were placed in an EasySep magnet for 10 min before pouring out to discard unbound solution. Magnetic latex bead-containing phagosomes and FeDex-containing lysosomes attached to the side of the tube were washed and resuspended with 1 ml homogenization buffer and spun down in a 1.5 ml Eppendorf tube at 16,000 × *g* for 15 min. Pellets were resuspended with lysis buffer and stored at −80 °C. Magnetic beads were removed by centrifugation prior to mass spectrometry.

### Mass spectrometry

All sample preparation, LC-MS/MS data collection, and MS data searches were carried out at the SPARC BioCentre at The Hospital for Sick Children (Toronto, Canada). Samples were reduced with DTT (10 mM, 60 °C, 1 h), alkylated with iodoacetamide (20 mM, room temperature, 45 min, dark), and digested overnight at 37 °C with trypsin (2 µg; Pierce). Peptides were dried by vacuum centrifugation, desalted on C18 ziptips (Millipore) using a DigestPro MSi (Intavis Bioanalytical Instruments), and dried again by vacuum centrifugation before resuspension in Buffer A (0.1% formic acid). Samples were analyzed by liquid chromatography-tandem mass spectrometry (LC-MS/MS) using an EASY-nanoLC 1200 system with a 1 h analysis and an Orbitrap Fusion Lumos Tribrid Mass Spectrometer (Thermo Fisher Scientific). The LC portion of the analysis consisted of an 18 min linear gradient running 3–20% of Buffer A to Buffer B (0.1% FA, 80% acetonitrile), followed by a 31 min linear gradient running 20–35% of Buffer A to Buffer B, a 2 min ramp to 100% Buffer B and 9 min hold at 100% Buffer B, all at a flow rate of 250 nL min^−1^. Samples were loaded into a 75 µm × 2 cm Acclaim PepMap 100 Pre-column followed by a 75 µm × 50 cm PepMax RSLC EASY-Spray analytical column filled with 2 µM C18 beads (Thermo Fisher Scientific). MS1 acquisition resolution was set to 120,000 with an automatic gain control (AGC) target value of 4 × 10^5^ and maximum ion injection time (IT) of 50 ms for a scan range of *m/z* 375–1500. Monoisotopic precursor selection (MIPS) was determined at the peptide level with a global intensity threshold of 10,000 and only peptides with charge states of 2–7 were accepted, with dynamic exclusion set to 10 s. Isolation for MS2 scans was performed in the quadrupole with an isolation window of *m/z* 0.7. MS2 scans were performed in the ion trap with maximum ion IT of 10 ms, AGC target value of 1 × 10^4^, and higher-energy collisional dissociation (HCD) activation with an NCE of 30. MS raw files were analyzed using Proteome Discoverer (version 2.2.0.388) and fragment lists and GO annotation searched against the mouse UniProt Reference database (downloaded Sept. 20, 2019; 54953 entries). For both search algorithms, the parent and fragment mass tolerances were set to 10 ppm and 0.6 Da, respectively, and only complete tryptic peptides with a maximum of two missed cleavages were accepted. Protein abundance was compared between UT and IFNβ-treated phagosomes and lysosomes. Complete proteomic datasets were deposited in the MassIVE repository, accession # MSV000087014 [10.25345/C5V51C].

### Lysosome acidification

BMDMs were plated at 6 × 10^5^ cells per well in 12-well tissue culture plates with glass coverslips and treated with 2400 IU ml^−1^ IFNβ 24 h before treatment. Cells were pulsed with 10 kDa FITC-dextran, washed and chased for 1 h in RPMI medium containing 10% FBS and IFNβ. Glass coverslips were transferred to an imaging chamber containing HEPES-buffered RPMI. The cells were visualized with a Leica DM IRB microscope. Once a cell was found that had bound fluorophore-labeled dextran, pH measurements were performed by fluorescence ratiometric imaging using a filter wheel (Sutter Instruments) to rapidly alternate between excitation filters. The FITC-dextran was excited by light from an EXFO X-cite 120 lamp (ExFo Life Sciences) transmitted through the appropriate excitation filters and directed to the sample using a dichroic mirror. The emitted light was captured by a CCD camera (Cascade II; Photometrics) after passing through an emission filter. MetaFluor (MDS Analytical Technologies) software was used to control the filter wheel and camera. An in situ calibration was performed for each lysosome after measurement. Samples were bathed in K^+^-rich buffers ranging from pH 4.5–9.6 containing 10 μg ml^−1^ nigericin. Images were taken 5 min after the addition of each calibration solution to ensure equilibration to the desired pH. The resulting fluorescence intensity ratio was plotted as a function of pH and fitted to a Boltzmann sigmoid that was used to interpolate ratios measured of lysosome acidification.

### DQ-BSA degradation

BMDMs were plated at 1 × 10^5^ cells per well in µ-Slide 8 Well IBIDI chambers and treated with 2400 IU ml^−1^ IFNβ 24 h before infection. 4 h prior to infection, cells were pulsed with green DQ-BSA (10 μg ml^−1^) for 1 h and chased with RPMI medium containing 10% FBS and IFNβ for 3 h. Cells were infected with 1:200 RFP-*E. coli* in RPMI. At 60 min p.i., all cells were washed three times with PBS+ and cultured in RPMI medium containing 10% FBS, IFNβ and 50 μg ml^−1^ gentamicin. 4 h p.i., cells were imaged live using a spinning disk confocal microscope equipped with a ×63 objective. DQ-BSA-positive bacteria were quantified using Volocity 6 software.

### *actA*:GFP *Lm* protein expression

J774 murine macrophage-like cells were plated at 2 × 10^6^ cells per well in 6-well tissue culture plates overnight in DMEM. Cells were infected with mid-log bacterial cultures resuspended 1:20 in 100 μl DMEM for 10 min. Infected cells were washed with PBS+ after 60 min and replaced with DMEM containing 50 μg ml^−1^ gentamicin. At each time point, cells were lysed with 0.1% Triton X-100 in PBS+, bacterial titers in the lysates were determined by plating dilutions for CFU of bacteria recovered via centrifugation. Bacteria were normalized to CFU and whole-cell lysates were generated by 3 mg ml^−1^ lysozyme treatment in Tris-EDTA for 60 min at 37 °C and subjected to western blot analysis.

### ActA, InlB, and LLO protein expression

WT and IFITM3 KO BMDMs were plated at 2.5 × 10^6^ cells per well in 6-well tissue culture plates and treated with 2400 IU ml^−1^ IFNβ 24 h before infection. Cells were infected with WT, TKO or Δ*actAplcAplcB Lm* at an MOI of 10 in RPMI. At 60 min p.i., all cells were washed three times with PBS+ and cultured in RPMI medium containing 10% FBS, 50 μg ml^−1^ gentamicin IFNβ. Cells were lysed with 1% Triton X-100 in 1 ml PBS+ at 1, 2, and 4 h p.i. 100 µl of lysate was used for serial dilution and plated on BHI agar plate to obtain bacterial CFU. Remaining lysates were spun down to recover soluble fraction for LLO and bacteria pellet for ActA and InlB. Samples were diluted and resuspended in sample buffer, boiled for 5 min, and subjected to western blot analysis.

### ActA degradation

RAW264.7 macrophages, WT and IFITM3 KO BMDMs were plated at 3 × 10^5^ cells per well in 24-well tissue culture plates. Where indicated, cells were treated with 2400 IU ml^−1^ IFNβ, 100 nM bafilomycin A, or protease inhibitors 24 h, 4 h and 30 min respectively before infection. Cells were infected with *actA*:GFP reporter strains; WT, Δ*hly Lm* and TKO *Lm* at an MOI of 10 in RPMI. At 60 min p.i., all cells were washed three times with PBS+ and cultured in RPMI medium containing 10% FBS, IFNβ and 50 μg ml^−1^ gentamicin. At 4 h p.i., cells were fixed with 2.5% PFA for 10 min. To visualize bacteria surface protein degradation in phagosomes, cells were stained with antibodies to ActA and LAMP1. Cells were imaged using a spinning disk confocal microscope equipped with a ×63 objective. ActA- and LAMP1-positive *actA*:GFP reporter-positive bacteria were quantified using Volocity 6 software.

### Actin recruitment

WT and IFITM3 KO BMDMs were plated at 3 × 10^5^ cells per well in 24-well tissue culture plates and treated with 2400 IU ml^−1^ IFNβ 24 h before infection. Cells were infected with WT *Lm* at an MOI of 20 in RPMI. At 60 min p.i., all cells were washed three times with PBS+ and cultured in RPMI medium containing 10% FBS, 50 μg ml^−1^ gentamicin IFNβ. At 1, 1.25, 1.5, 1.75, 2 and 4 h p.i., cells were fixed with 2.5% PFA. Differential antibody staining was carried out to stain extracellular bacteria before permeabilizing the cells to stain for total bacteria. BMDMs were also stained for actin using phalloidin. Cells were imaged on a Quorum spinning disk confocal scan head equipped with a ×63 objective. Actin recruitment was quantified as phalloidin-positive internalized bacteria (total bacteria minus extracellular bacteria) over the time course, and motile *Lm* (actin comet tail-positive bacteria) were quantified 4 h p.i., using Volocity 6 software.

### Infection focus assay

Assay was conducted as previously described^57^. Briefly, WT and IFITM3 KO BMDMs were plated at 1.5 × 10^6^ cells per well in 24-well tissue culture plates and treated with 2400 IU ml^−1^ IFNβ 24 h before infection. AML12, MEF and Caco-2 cells were plated at 2, 4, and 2.5 × 10^5^ cells per well, respectively, in 24-well tissue culture plates and treated with 2400 IU ml^−1^ IFNβ 24 h before infection. Cells were infected with WT *Lm* at an MOI of 0.001 (BMDM), 1 (AML12), 0.5 (MEFs) or 0.1 (Caco-2) in RPMI (BMDM) or DMEM (all others). At 18 h p.i., cells were fixed and counterstained with *Lm* and DAPI, and imaged on a Quorum spinning disk confocal scan head equipped with a ×10 objective. The number of infected cells per focus of infection was quantified using Volocity 6 software.

### Replication assay

WT and IFITM3 KO BMDMs were plated at 3 × 10^5^ cells per well (sub-confluence density) in 24-well tissue culture plates and treated with 2400 IU ml^-1^ IFNβ 24 h before infection. Cells were infected with WT or Δ*actA Lm* at an MOI of 1 in RPMI. At 60 min p.i., all cells were washed three times with PBS+ and cultured in RPMI medium containing 10% FBS, 50 μg ml^−1^ gentamicin IFNβ. At 2, 4, 8 and 12 h p.i., cells were lysed with PBS+ containing 1% Triton X-100. Serial dilutions of the lysates were plated on BHI agar plates and incubated at 37 °C for 16 h for subsequent quantification of bacterial CFU.

### In vivo infection

8–12-week-old sex- and age-matched WT, IFITM3 KO, and IFNAR1 KO mice were used for WT *Lm* infection. Mice were restrained using Mouse Tail Illuminator Restrainer (Braintree Scientific), tail was swabbed with 70% EtOH and warmed to dilate lateral tail vein. Mice were infected with 5 × 10^4^ bacteria diluted in 200 µl of PBS+ via intravenous injection using 30 G x ½ needles (BD). Mice were monitored and weighed 3 times a day at 9 am, 3 pm and 9 pm. Survival rate was plotted using Kaplen–Meier function after 10 days p.i. Organs used to determine bacterial load were harvested from a separate group of mice 72 h p.i. Mice were euthanized by CO_2_, and liver, spleen and brain were obtained and homogenized in 1 ml PBS+, and serial dilutions of the tissue were plated on BHI agar plates and incubated at 37 °C for 16 h for subsequent quantification of CFU. Final bacterial load was calculated after normalization to tissue weight.

### Tissue staining

Liver obtained from WT and IFITM3 KO mice infected with WT *Lm* or GFP-*Lm* for 72 h was embedded in OCT medium (Tissue-Tek #4583) and snap-frozen in liquid nitrogen. 5 µm sections collected in 4 series were obtained using a Cryostat (Leica CM 1850) and mounted on charged slides (VWR #CA48311-703). Tissue was fixed in ice-cold methanol for 10 min at −20 °C. For hematoxylin and eosin staining, slides were rehydrated and stained according to the following: Hematoxylin (3 min), H_2_O (1 min), Clarifier (1 min), H_2_O (1 min), Bluing Reagent (3 min), H_2_O (1 min), 95% EtOH (rinse), Eosin (30 s), 75% EtOH (rinse), 95% EtOH (rinse), 100% EtOH (1 min ×3), Xylene (1 min ×3). Tissue was sealed with mounting media (Sigma #107960) and liver infection foci were imaged using Leica DM1000LED with a ×20 objective. Number of foci were calculated per square mm of tissue. For immunofluorescence staining, slides were blocked with 5% BSA, 10% goat serum and 0.1% Triton X-100 diluted in PBS+ for 30 min at RT. Tissue were stained with antibody to *Lm*, ActA, CD3e and F4/80 overnight at 4 °C, washed with PBS+ and counterstained with secondary antibody and DAPI. Tissue was imaged on a Quorum spinning disk confocal scan head equipped with a ×10 and ×60 objective. ActA and immune cell recruitment, and the number of infected cells per focus of infection were quantified using Volocity 6 software.

### IFNβ production

WT and IFITM3 KO BMDMs were plated at 4 × 10^6^ cells per well in 6-well tissue culture plates and treated with 2400 IU ml^−1^ IFNβ 24 h before infection. Cells were infected with WT Lm at an MOI of 0.001 in RPMI. At 60 min p.i., all cells were washed three times with PBS+ and cultured in RPMI medium containing 10% FBS and 50 μg ml^−1^ gentamicin. Cell culture supernatant was collected 4, 8, 12 and 24 h p.i. IFNβ levels were measured using ELISA kit (PBL Assay Science #4200-1).

### RNA isolation and qPCR

Liver tissue from WT and IFITM3 KO mice infected with WT *Lm* was obtained and RNA was isolated using the RNeasy kit (QIAGEN #74104). cDNA was synthesized using iScript Reverse Transcription Supermix for reverse transcription. 10 ng of cDNA per reaction was used for quantitative PCR using SsoFast EvaGreen Supermix (Bio-Rad #1708840) with Hprt as the housekeeping gene. The following primers were obtained from Sigma: *Ifnb* (F-CCCTATGGAGATGACGGAGA R-CTGTCTGCTGGTGGAGTTCA), *Ifngr1* (F-GTAGCCTCACCGCCTATCAC R-GGGCCTCTCCTGTGAGTCTA), *Tnfa* (F-CCCATGGATGTCCCATTTAG R-CAATCAGGAGGGTGTGTGTG), *Il4* (F-GCCTGCTTTTCACATGAGGT R-AAATATGCGAAGCACCTTGG), *Il10* (F-CCAAGCCTTATCGGAAATGA R-TTTTCACAGGGGAGAAATCG), *Il12* (F-AGCATAAGAGACGCCCTCAA R-GGTGTTACAGGCCCAAAGAA). All primers used are listed in Supplementary Table 1.

### Statistical analysis

Statistical analyses and graph plotting were conducted using GraphPad Prism v.8.4.1. The mean ± s.d. is shown in figures, and *P* values were calculated as described in figure legends. *P* < 0.05 was considered statistically significant. Unless stated otherwise, *P < 0.05, **P < 0.01, ***P < 0.001, ****P < 0.0001, not significant (ns) *P* > 0.05.

### Reporting summary

Further information on research design is available in the Nature Research Reporting Summary linked to this article.

## Supplementary information

Supplementary Information

Reporting Summary

## Data Availability

The data that support the findings of this study are available from the corresponding authors upon request. Complete proteomic datasets were deposited in the MassIVE repository, accession # MSV000087014 [10.25345/C5V51C]. Source data are provided with this paper.

## Source data

Source Data
