# Supplementary material for: Listeria exploits IFITM3 to suppress antibacterial activity in phagocytes
Source: Nat Commun. 2021 Aug 17;12:4999. doi: 10.1038/s41467-021-24982-0 (PMC8371165; doi:10.1038/s41467-021-24982-0)
Supplement: Supplementary file 1 — Supplementary Information [file 41467_2021_24982_MOESM1_ESM.pdf]

Supplementary Information

Listeria exploits IFITM3 to suppress antibacterial activity in phagocytes

Joel M.J. Tan[1,2], Monica E. Garner[1,2], James M. Regeimbal[3], Catherine J. Greene[4], Jorge D. Rojas Márquez[1], Dustin A. Ammendolia[1,5], Adam R.R. McCluggage[1], Taoyingnan Li[1,5], Katherine J. Wu[3], Marija Cemma[1,5], Philip P. Ostrowski[1,6], Brian Raught[7,8], Michael S. Diamond[9], Sergio Grinstein[1,2,6], Robin M. Yates[4], Darren E. Higgins[3,Ψ,*], John H. Brumell[1,2,5,10,Ψ,*]

1. Cell Biology Program, Hospital for Sick Children, Toronto, ON, Canada, M5G 0A4

2. Institute of Medical Science, University of Toronto, Toronto, ON, Canada, M5S 1A1

3. Department of Microbiology, Blavatnik Institute, Harvard Medical School, Boston, MA, USA 02115

4. Department of Biochemistry and Molecular Biology, University of Calgary, Calgary, AB, Canada, T2N 4N1

5. Department of Molecular Genetics, University of Toronto, Toronto, ON, Canada, M5S 1A1

6. Department of Biochemistry, University of Toronto, Toronto, ON, Canada, M5S 1A1

7. Princess Margaret Cancer Centre, University Health Network, Toronto, ON, M5S 1A8, Canada

8. Department of Medical Biophysics, University of Toronto, Toronto, ON M5S 1A8, Canada

9. Departments of Medicine, Molecular Microbiology, Pathology & Immunology, Washington University School of Medicine, St. Louis, MO, USA 63110

10. SickKids IBD Centre, Hospital for Sick Children, Toronto, ON, Canada, M5G 0A4

[Ψ]Jointly supervising authors

*Correspondence:
John H. Brumell, Cell Biology Program, Hospital for Sick Children, 686 Bay Street PGCRL, Toronto, ON, Canada M5G 0A4, Tel: 416-813-7654 ext. 303555. E-mail: john.brumell@sickkids.ca

Darren E. Higgins, Department of Microbiology, Blavatnik Institute, Harvard Medical School, NRB Room 854, 77 Avenue Louis Pasteur, Boston, MA, USA 02115, Tel: 617-432-4156, E-mail: dhiggins@hms.harvard.edu

Supplementary Figures

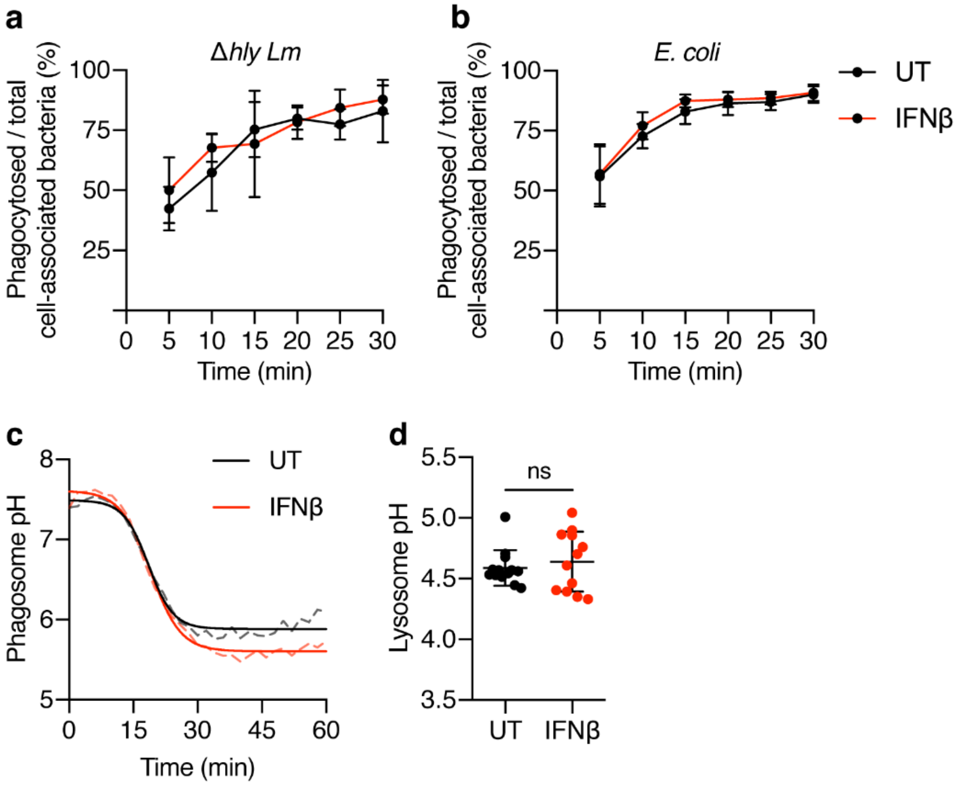

Supplementary Fig. 1 | Type I IFN does not affect phagocytosis or acidification. Number of internalized Δ*hly Lm* (**a**) and *E. coli* (**b**) in BMDMs untreated (UT) or treated with IFNβ were enumerated over a 30 min time course. Differential antibody staining was carried out to determine the population of intracellular versus extracellular bacteria. Phagosome (**c**) and lysosome (**d**) acidification in BMDMs treated with or without IFNβ fed with carboxyfluorescein succinimidyl ester (CFSE)-particle (phagosome) or FITC-dextran (lysosome). Fluorescence ratiometric imaging was used to determine pH value of phagosomes (over 60 min) and lysosomes (at 60 min) calibrated against a standard curve. Solid lines represent nonlinear regression fit of the dashed lines. Data shown are means ± standard deviation (s.d.) for n = 3 independent experiments. *P* value was calculated using two-tailed unpaired t-test in (**d**). ns, not significant, *P* = 0.5348.

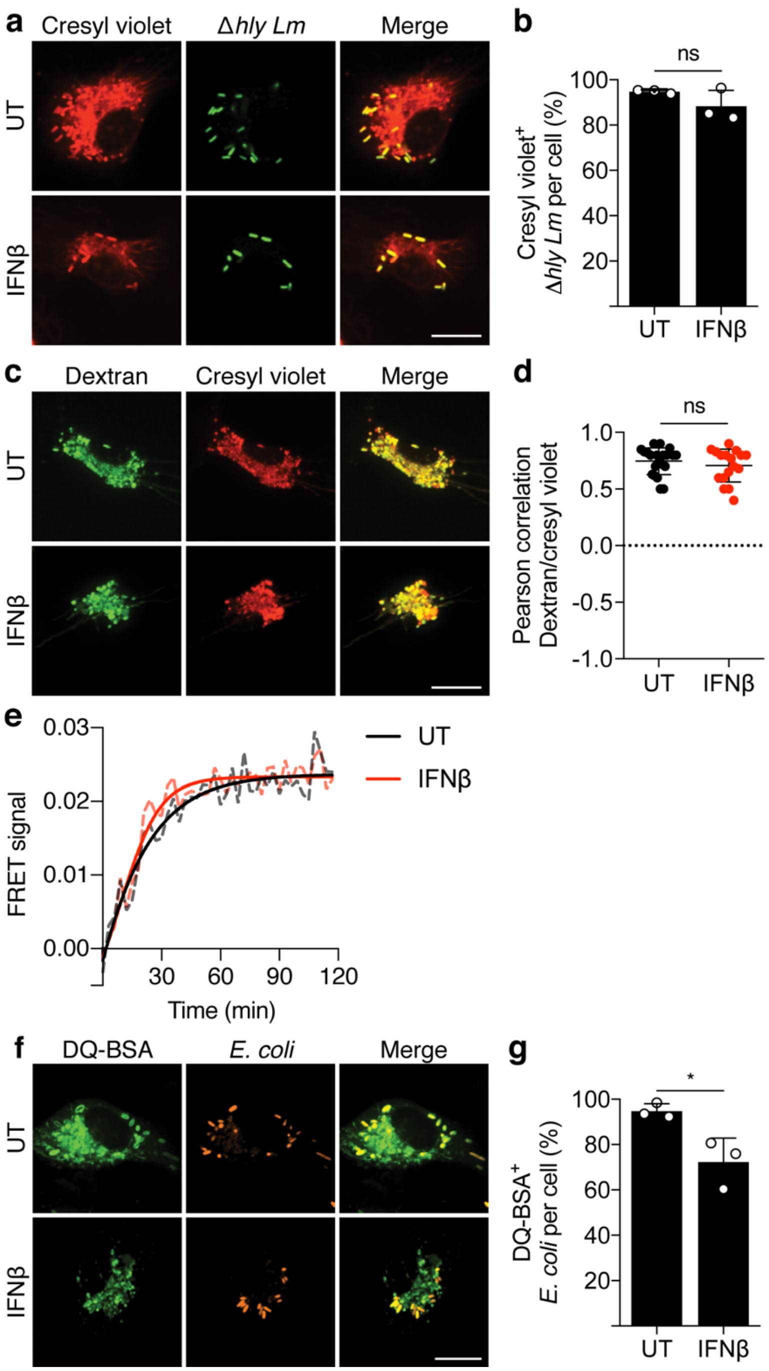

Supplementary Fig. 2 | Effect of type I IFN on early phagosome maturation events. a, b, Representative images (**a**) and quantification (**b**) of Δ*hly Lm* in lysosomes of BMDMs treated with or without IFNβ 4 h post-infection (p.i.) using lysosomal marker cresyl violet after 30 min**. c, d,** Endolysosomal fusion measured in dextran-fed BMDMs treated with or without IFNβ using lysosomal marker cresyl violet after 30 min. Representative images (**c**) and Pearson correlation (**d**) of colocalization between dextran and cresyl violet-positive compartments. **e,** Phagolysosomal fusion measured using FRET-based assay in BMDMs treated with or without IFNβ using particles labeled with donor fluorophore (phagosomes) and acceptor fluorophore (lysosomes) over 2 h. Solid lines represent nonlinear regression fit of the dashed lines. **f, g,** Representative images (**f**) and quantification (**g**) of phagosomal proteolysis measured using self-quenched substrate DQ-BSA that fluoresces in the presence of proteases. *E. coli* residing in DQ-BSA-positive compartments in BMDMs treated with or without IFNβ were enumerated after 4 h. Data shown are means ± s.d. for at least n = 3 independent experiments. *P* value (**b, d, g**) was calculated using two-tailed unpaired *t*-test. *P = 0.0252. ns, not significant, $P > 0.1893$. Scale bars, 11 µm.

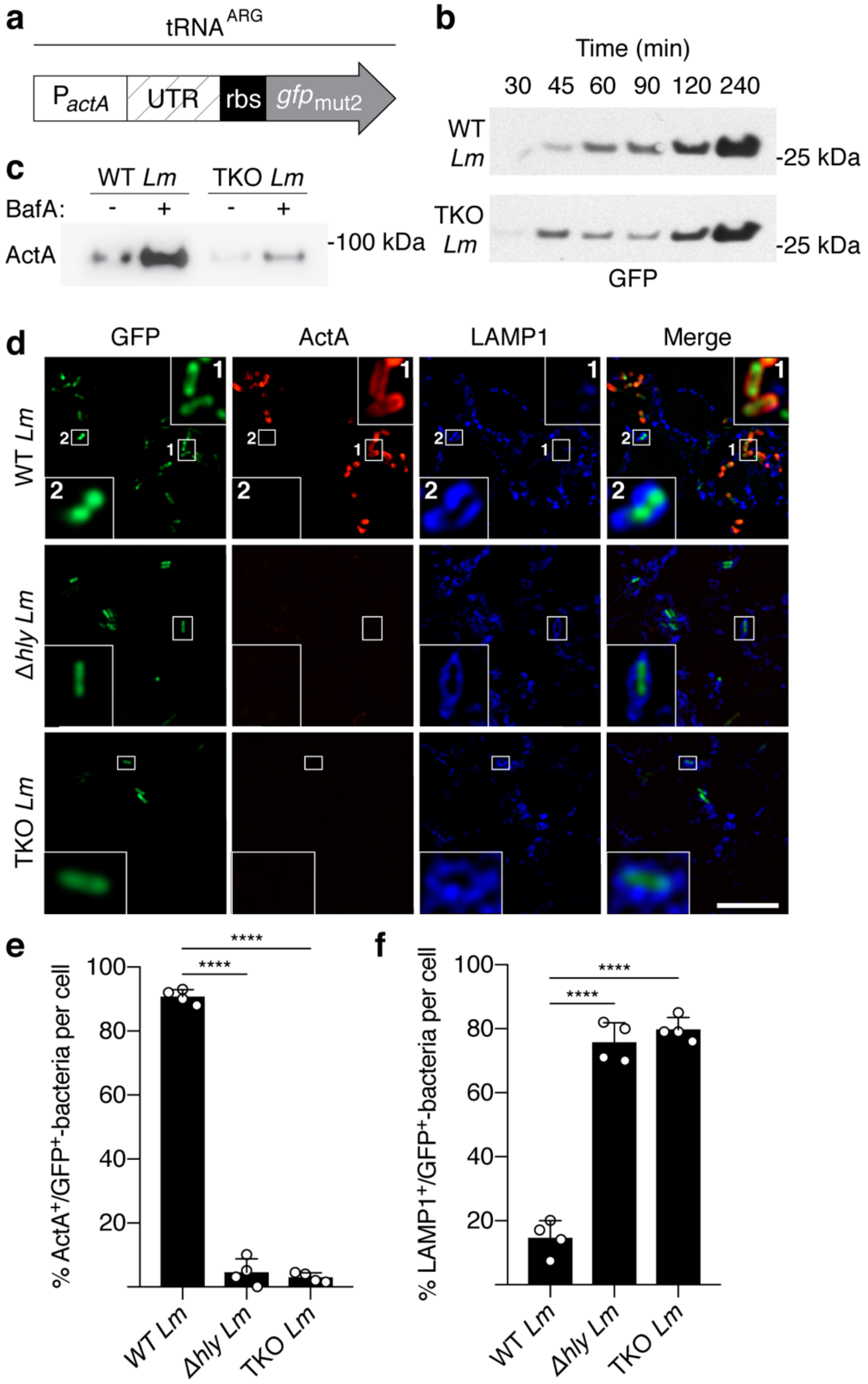

Supplementary Fig. 3 | *Lm* expresses ActA within phagosomes. a, Schematic of GFP reporter fusion construct separately integrated in single copy at the tRNA^{ARG} locus of WT *Lm* 10403S strain. P*actA* promoter with the full-length *actA* 5' untranslated region (UTR) and ribosome binding site (rbs). **b,** Representative images of the production of *actA*:GFP measured by Western blot in J774 macrophages infected with indicated GFP reporter strains over 4 h. **c,** Representative image of the production of ActA measured by Western blot in BMDMs treated with and without bafilomycin A infected with indicated strains 4 h p.i. **d-f,** Representative images (**d**) and quantification of ActA-positive bacteria expressing *actA*:GFP (**e**) or *actA:*GFP-positive bacteria in LAMP1-positive compartments (**f**) in RAW macrophages infected with indicated strains 4 h p.i. Data and images shown are means ± s.d. for at least n = 3 independent experiments. *P* value was calculated using one-way ANOVA. *****P* < 0.0001. Scale bar, 10 µm. TKO, triple knockout mutant strain Δ*hlyplcAplcB Lm*. Source data are provided as a Source Data file.

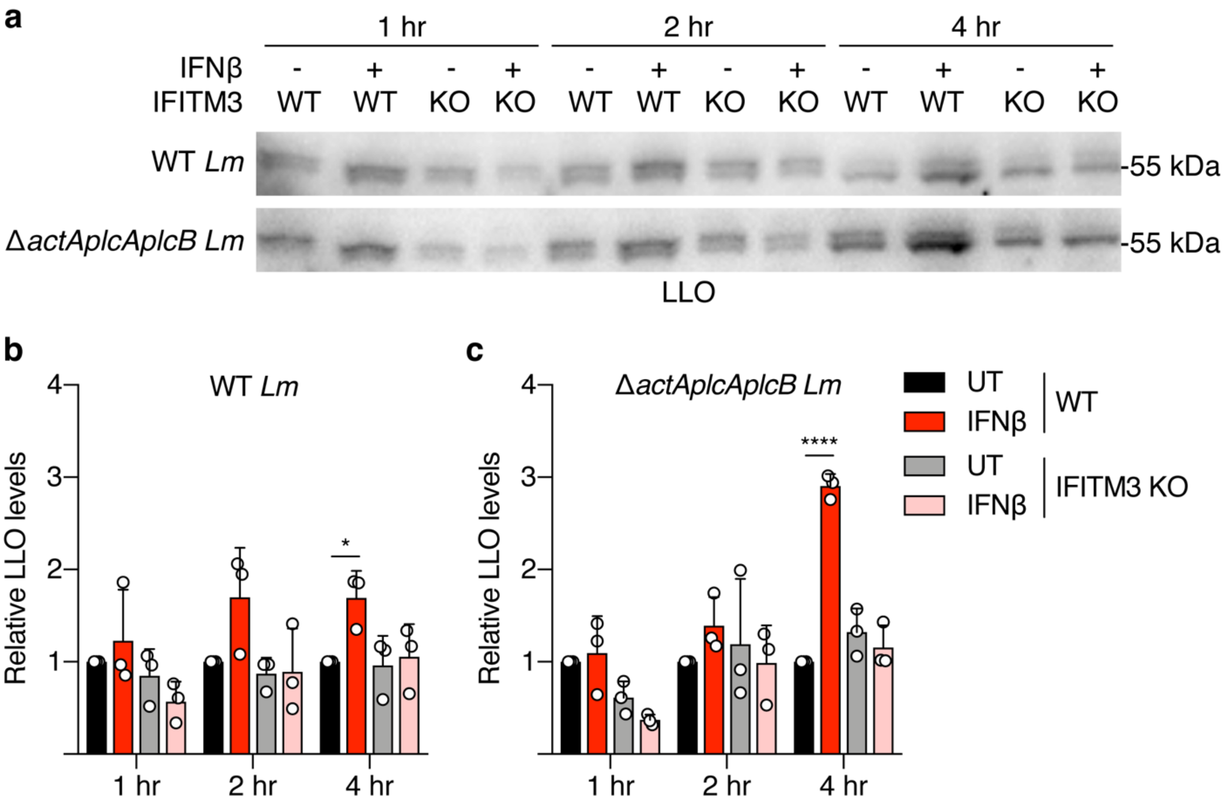

Supplementary Fig. 4 | IFITM3 suppresses LLO degradation in phagosomes. a-c, Representative images (**a**) and quantification of LLO production measured by Western blot in IFITM3 WT and KO BMDMs infected with (**b**) WT *Lm* or (**c**) *actAplcAplcB Lm* over 4 h. Data shown are means ± standard deviation (s.d.) for n = 3 independent experiments. *P* value was calculated using two-way ANOVA. *$P = 0.0164$, ****$P < 0.0001$. Source data are provided as a Source Data file.

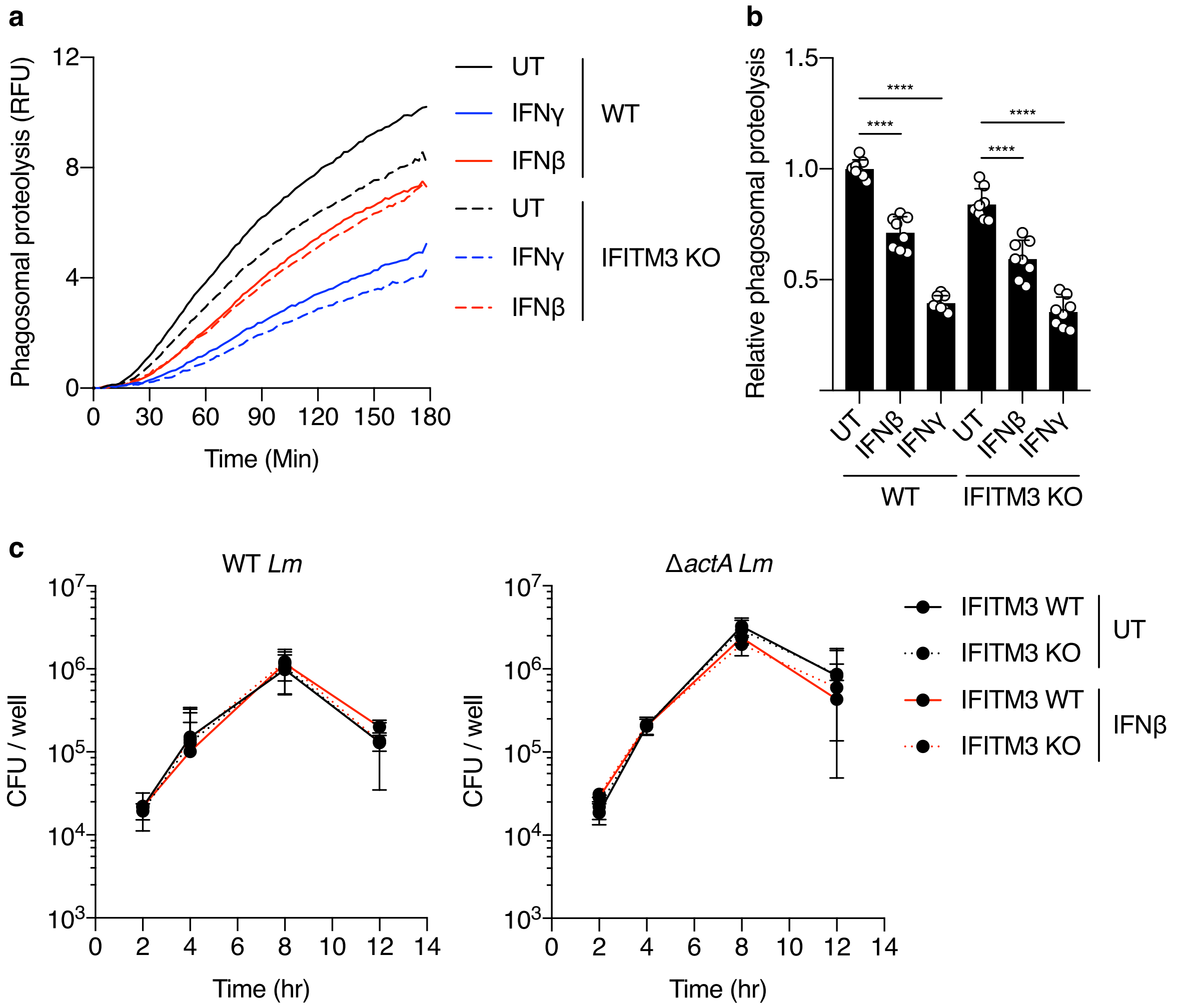

Supplementary Fig. 5 | Effect of IFITM3 on bulk proteolysis and *Lm* growth in the cytosol. a, b, Bulk proteolysis of DQ-BSA-labelled particles in phagosomes treated with IFNβ or IFNγ, in IFITM3 WT and KO BMDMs. Slope of the curve's linear portion in (**a**) is displayed in (**b**). **c,** Growth curve of IFITM3 WT and KO BMDMs infected with WT or Δ*actA Lm* over 12 h. Cells were seeded at sub-confluence density and infected with an MOI of 1. Data shown are means ± standard deviation (s.d.) for at least n = 3 independent experiments. *P* value was calculated using one-way ANOVA. ****$P < 0.0001$.

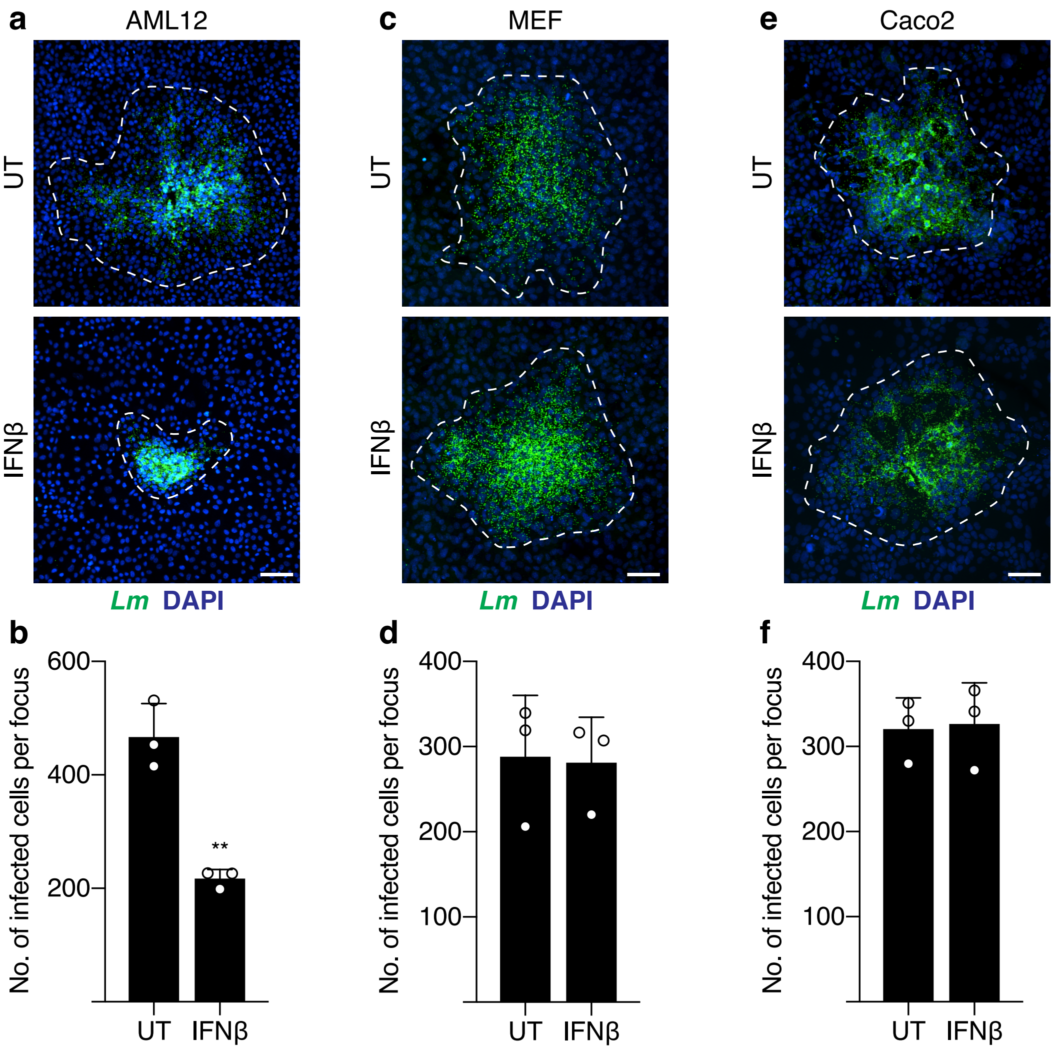

Supplementary Fig. 6 | Type I IFN does not promote *Lm* cell-to-cell spread in AML12, MEF or Caco2 cells. a-f, Representative images (**a, c, e**) and quantification (**b, d, f**) of an infection focus assay to measure WT *Lm* cell-to-cell spread. Indicated cell types were treated with or without IFNβ and infected for 18 h. Dotted lines delineate edge of the infection foci. Scale bars, 70 µm. Data shown are means ± s.d. for n = 3 independent experiments. *P* value was calculated using two-way ANOVA. $^{**}P = 0.0021$.

Supplementary Table

Primer	Sequence
221	AAGTCGACTTATTTGTATAGTTCATCCATGCCATG
432	CGCGGCCGGTGGTATCCCGAATAAAGCAGCC
434	GAAAAGTTCTTCTCCTTTACTCATTTATACTCCCTCCTCGTGATAC
435	GTATCACGAGGAGGGAGTATAAATGAGTAAAGGAGAAGAACTTTTC
Ifnβ	F-CCCTATGGAGATGACGGAGA R-CTGTCTGCTGGTGGAGTTCA
Ifnγr1	F-GTAGCCTCACCGCCTATCAC R-GGGCCTCTCCTGTGAGTCTA
Tnfα	F-CCCATGGATGTCCCATTTAG R-CAATCAGGAGGGTGTGTGTG
Il4	F-GCCTGCTTTTCACATGAGGT R-AAATATGCGAAGCACCTTGG
Il10	F-CCAAGCCTTATCGGAAATGA R-TTTTCACAGGGGAGAAATCG
Il12	F-AGCATAAGAGACGCCCTCAA R-GGTGTTACAGGCCCAAAGAA

Supplementary Table 1 | Summary of primers used to generate *Lm* strains, and primers used for qPCR.
