# Supplementary material for: Listeria exploits IFITM3 to suppress antibacterial activity in phagocytes
Source: Nat Commun. 2021 Aug 17;12:4999. doi: 10.1038/s41467-021-24982-0 (PMC8371165; doi:10.1038/s41467-021-24982-0)
Supplement: Supplementary file 2 — Reporting Summary [file 41467_2021_24982_MOESM2_ESM.pdf]

nature research

Corresponding author(s): J.H.B and D.E.H

Last updated by author(s): Jul 3, 2021

Reporting Summary

Nature Research wishes to improve the reproducibility of the work that we publish. This form provides structure for consistency and transparency in reporting. For further information on Nature Research policies, see Authors & Referees and the Editorial Policy Checklist .

Statistics

For all statistical analyses, confirm that the following items are present in the figure legend, table legend, main text, or Methods section.

n/a	Confirmed	
☐	☒	The exact sample size (*n*) for each experimental group/condition, given as a discrete number and unit of measurement
☐	☒	A statement on whether measurements were taken from distinct samples or whether the same sample was measured repeatedly
☐	☒	The statistical test(s) used AND whether they are one- or two-sided<br>*Only common tests should be described solely by name; describe more complex techniques in the Methods section.*
☒	☐	A description of all covariates tested
☒	☐	A description of any assumptions or corrections, such as tests of normality and adjustment for multiple comparisons
☐	☒	A full description of the statistical parameters including central tendency (e.g. means) or other basic estimates (e.g. regression coefficient) AND variation (e.g. standard deviation) or associated estimates of uncertainty (e.g. confidence intervals)
☐	☒	For null hypothesis testing, the test statistic (e.g. *F*, *t*, *r*) with confidence intervals, effect sizes, degrees of freedom and *P* value noted<br>*Give P values as exact values whenever suitable.*
☒	☐	For Bayesian analysis, information on the choice of priors and Markov chain Monte Carlo settings
☒	☐	For hierarchical and complex designs, identification of the appropriate level for tests and full reporting of outcomes
☒	☐	Estimates of effect sizes (e.g. Cohen's *d*, Pearson's *r*), indicating how they were calculated

Our web collection on statistics for biologists contains articles on many of the points above.

Software and code

Policy information about availability of computer code

Data collection	Images were collected on a Quorum spinning disk confocal scan head. Western blot was imaged using Bio-Rad ChemiDoc system. Phagosome measurements were collected using FLUOstar Optima fluorescent plate reader. Mass spectrometry was done using EASY-nanoLC 1200 system with a 1 h analysis and an Orbitrap Fusion Lumos Tribrid Mass Spectrometer.
Data analysis	Confocal microscopy images were analyzed using Volocity 6 software, and imported into Adobe Photoshop and assembled in Adobe Illustrator for labelling. Densitometry was performed using ImageJ software. Statistical analyses and graph plotting were conducted using GraphPad Prism v.8.4.1.

For manuscripts utilizing custom algorithms or software that are central to the research but not yet described in published literature, software must be made available to editors/reviewers. We strongly encourage code deposition in a community repository (e.g. GitHub). See the Nature Research guidelines for submitting code & software for further information.

Data

Policy information about availability of data

All manuscripts must include a data availability statement. This statement should provide the following information, where applicable:

- Accession codes, unique identifiers, or web links for publicly available datasets
- A list of figures that have associated raw data
- A description of any restrictions on data availability

The data that support the findings of this study are available from the corresponding authors upon request. Complete proteomic datasets were deposited in the MassIVE repository, accession # MSV000087014 [doi.org/doi:10.25345/C5V51C].

Field-specific reporting

Please select the one below that is the best fit for your research. If you are not sure, read the appropriate sections before making your selection.

☒ Life sciences ☐ Behavioural & social sciences ☐ Ecological, evolutionary & environmental sciences

For a reference copy of the document with all sections, see nature.com/documents/nr-reporting-summary-flat.pdf

Life sciences study design

All studies must disclose on these points even when the disclosure is negative.

Sample size	No sample size calculation was performed, sample sizes were chosen based common practice in the field and the variability within an experiment (Czuczman et al., 2014).
Data exclusions	No data was excluded from analysis.
Replication	Findings were reproduced at least 3 times with biological replicates.
Randomization	Randomization was not relevant to in vitro experiments since more than one cell type was used. In vivo mice experiments had to be sex- and age-matched between WT and KO mice to ensure reproducibility and minimal variability.
Blinding	Blinding was not relevant to in vitro experiments since cells were treated with more than one condition. Blinding does not affect data outcome of in vivo infections as every mice is given the same treatment.

Reporting for specific materials, systems and methods

We require information from authors about some types of materials, experimental systems and methods used in many studies. Here, indicate whether each material, system or method listed is relevant to your study. If you are not sure if a list item applies to your research, read the appropriate section before selecting a response.

Materials & experimental systems

n/a	Involved in the study	
☐	☒	Antibodies
☐	☒	Eukaryotic cell lines
☒	☐	Palaeontology
☐	☒	Animals and other organisms
☒	☐	Human research participants
☒	☐	Clinical data

Methods

n/a	Involved in the study	
☒	☐	ChIP-seq
☒	☐	Flow cytometry
☒	☐	MRI-based neuroimaging

Antibodies

Antibodies used	Primary antibodies (1:100) used for immunofluorescence were rabbit anti-Lm (#B223021 from BD Biosciences), rabbit-anti-E. coli (#ab20640 from Abcam), rabbit-anti-ActA (gift from P. Lauer, Aduro BioTech), rabbit-anti-InlB (gift from M. Loessner, ETH Zurich), mouse-anti-p60 (#NBP2-80120 from Novus), rabbit-anti-LLO (#ab200538 from Abcam), rat-anti-F4/80 (#ab16911 from Abcam), biotin-anti-CD3e (#13-0031-85 from ThermoFisher), and rat anti-mouse LAMP1 (1D4B was deposited to the Developmental Studies Hybridoma Bank by J.T. August).
Validation	Antibodies were validated by the manufacturer or previous publication.<br><br>Lm (https://www.fishersci.ca/shop/products/bd-difco-i-listeria-i-o-antisera-3/df2302500)<br>E. coli (https://www.abcam.com/biotin-e-coli-antibody-ab20640.html)<br>ActA (Osborne et al., 2017)<br>InlB (Sumrall et al., 2021)<br>p60 (https://www.novusbio.com/products/listeria-monocytogenes-p60-antibody-p6007_nbp2-80120)<br>LLO (https://www.abcam.com/listeriolysin-llo-antibody-ab200538.html)<br>F4/80 (https://www.abcam.com/f480-antibody-bm8-ab16911.html)<br>CD3e (https://www.thermofisher.com/antibody/product/CD3e-Antibody-clone-145-2C11-Monoclonal/13-0031-82)<br>LAMP1 (https://dshb.biology.uiowa.edu/1D4B)

Eukaryotic cell lines

Policy information about cell lines

Cell line source(s)	RAW 264.7 and J774 macrophages, AML12, MEF, L929, Caco2 were purchased from American Type Culture Collection (ATCC, Rockville, MD). Bone marrow derived macrophages were obtained as described in Methods.
Authentication	All cells from ATCC were authenticated by ATCC and SickKids Biobank. Bone marrow derived macrophages were confirmed by western blotting and PCR. No further authentication procedures were performed.
Mycoplasma contamination	All cell lines tested negative for mycoplasma prior to experimentation.
Commonly misidentified lines (See ICLAC register)	No commonly misidentified cell lines were used in this study.

Animals and other organisms

Policy information about studies involving animals; ARRIVE guidelines recommended for reporting animal research

Laboratory animals	B6.129S2-Ifnar1-/- (IFNAR1 KO; Jackson stock #32045-JAX) and B6(Cg)-Tyrc-2J-Ifitm3-/- (IFTIM3 KO; obtained from M. Diamond, backcrossed >10 generations with C57BL/6J at Washington University at St. Louis) mice were bred in house at the Hospital for Sick Children Animal Care Facility. C57BL/6J (stock #000664) and B6(Cg)-Tyrc-2J (stock #000058) mice, originally from The Jackson Laboratory, were also bred in house and used as controls. All experiments were performed with 6–12-week-old sex- and age-matched male and female mice that were maintained on a 12 h light–dark cycle, with food and water available ad libitum. All animals were bred separately and housed in specific pathogen-free barrier rooms kept at 22-26°C and 45-55% humidity.
Wild animals	No wild animals were used in this study.
Field-collected samples	No field-collected samples were used in this study.
Ethics oversight	All experiments described in this study were carried out in accordance with the Guide for the Humane Use and Care of Laboratory Animals and ethical approval was obtained from The Hospital for Sick Children's Animal Care Committee (AUP #52111).

Note that full information on the approval of the study protocol must also be provided in the manuscript.
